# Research utility and limitations of textual data in the National Violent Death Reporting System: a scoping review and recommendations

**DOI:** 10.1186/s40621-023-00433-w

**Published:** 2023-05-09

**Authors:** Linh N. Dang, Eskira T. Kahsay, LaTeesa N. James, Lily J. Johns, Isabella E. Rios, Briana Mezuk

**Affiliations:** 1 Department of Epidemiology, Center for Social Epidemiology and Population Health, University of Michigan School of Public Health, 1415 Washington Heights, Ann Arbor, MI 48109 USA; 2 Taubman Health Sciences Library, University of Michigan, Ann Arbor, MI USA; 3 Department of Epidemiology, University of Michigan School of Public Health, Ann Arbor, MI USA

**Keywords:** National Violent Death Reporting System, Injury, Violence, Epidemiology, Scoping review, Suicide, Homicide, Qualitative data, Data science

## Abstract

**Background:**

Many studies of injury deaths rely on mortality data that contain limited contextual information about decedents. The National Violent Death Reporting System (NVDRS) is unique among such data systems in that each observation includes both quantitative variables and qualitative texts (called “narratives”) abstracted from original source documents. These narratives provide rich data regarding salient circumstances that can be used to inform prevention efforts. This review provides a comprehensive summary of peer-reviewed research using NVDRS narratives over the past 20 years, including the limitations of these texts and provides recommendations on utilizing and improving narrative quality for researchers and practitioners.

**Main body:**

Studies that used narratives to examine deaths related to suicide, homicide, undetermined intent, accidental firearm, or legal intervention were identified by a title/abstract screening, followed by a full-text review. The search was conducted on English-language, peer-reviewed literature and government reports published from 2002 to 2022 in PubMed, PsycInfo, Scopus, and Google Scholar. Abstracted elements focused on the methodologies used to analyze the narratives, including approaches to explore potential biases in these texts. Articles were abstracted independently by two reviewers, with disagreements resolved through consensus discussion. During the 20-year period, 111 articles used narratives. Two-thirds studied suicide (*n* = 48, 43%) and homicides (*n* = 25, 23%). Most studies analyzed the narratives using manual review (*n* = 81, 73%) and keyword searches (*n* = 9, 8%), with only 6 (5%) using machine learning tools. Narratives were mainly used for case finding (*n* = 49, 44%) and characterization of circumstances around deaths (*n* = 38, 34%). Common challenges included variability in the narratives and lack of relevant circumstantial details for case characterization.

**Conclusion:**

Although the use of narratives has increased over time, these efforts would be enhanced by detailed abstraction of circumstances with greater salience to injury research and prevention. Moreover, researchers and practitioners would benefit from guidance on integrating narratives with quantitative variables and standardized approaches to address variability in the completeness and length of narratives. Such efforts will increase the reliability of findings and set the stage for more widespread applications of data science methods to these texts.

**Supplementary Information:**

The online version contains supplementary material available at 10.1186/s40621-023-00433-w.

## Background

Violent deaths are a significant public health burden in the USA, with over 270,000 deaths attributed to fatal injury in 2020 (Centers for Disease Control and Prevention 2021a). Evidence-based violence prevention efforts have been hampered historically by a lack of high quality and timely surveillance data on these deaths and their circumstances. Calls for a national fatal intentional injury system that tracked these deaths resulted in collaborative efforts to create such a monitoring system (Barber et al. 2013; Hemenway et al. 2009), which began as the National Violent Injury Statistics System (NVISS). The National Violent Death Reporting System (NVDRS, publicly available at https://www.cdc.gov/violenceprevention/datasources/nvdrs/dataaccess.html), implemented by the Centers for Disease Control (CDC) in 2002, arose from this ongoing effort as a federally funded, active state-based reporting system that collects data on violent deaths, defined as “death that results from the intentional use of physical force or power, threatened or actual, against oneself, another person, or a group or community” (Centers for Disease Control and Prevention 2022b). These include suicide, homicide, legal intervention deaths, unintentional firearm deaths, and deaths with undetermined intent.

The NVDRS- and state-specific Violent Deaths Reporting Systems (VDRS) collect and link primary investigative information from a number of existing sources, including death certificates, coroners and medical examiners (C/ME), toxicology records, and law enforcement (LE) reports, to create the most comprehensive, centralized surveillance reporting system of violent deaths. The NVDRS also incorporates secondary sources of information from crime labs, hospitals, court records, press releases, and Intimate Partner Violence (IPV) and Child Fatality Review (CFR) reports (Centers for Disease Control and Prevention 2022b). The scope and methodology of the NVDRS has been described in additional detail elsewhere (Centers for Disease Control and Prevention 2022b; Blair et al. 2016b; Steenkamp et al. 2006; Paulozzi 2004). As of 2018, the NVDRS expanded to all 50 US states, Puerto Rico, and the District of Columbia. This reporting system has substantial potential to inform policy and prevention practice, with examples of this already demonstrated in various states (Powell et al. 2006).

Beyond this publicly available data, the CDC manages a centralized Restricted Access Database of the NVDRS (RAD-NVDRS) which includes additional variables encompassing decedent and suspect demographic variables, incident circumstance variables, and toxicology variables. Notably, the RAD-NVDRS contains short text narratives (between 150 and 300 words) written by VDRS staff using C/ME and LE reports, suicide notes, and interviews with the decedents’ family/friends (Centers for Disease Control and Prevention 2022b). These narratives provide a rich source of qualitative data to supplement the NVDRS’s existing quantitative variables. In addition to validating coding decisions on coded variables, the narratives provide opportunities to identify emerging and novel risk factors salient to violent deaths beyond existing quantitative variables in the NVDRS. They can also be used to identify violent deaths that are often difficult to accurately count, such as accidental gun deaths (Barber and Hemenway 2011) and homicides by police (Barber et al. 2016). A growing number of studies have used the NVDRS to investigate epidemiologic trends, precipitating factors, and contextual factors of violent deaths as well as how these correlates vary by race/ethnicity, occupation, and physical and mental health (Mezuk et al. 2021).

Although the narratives serve as a valuable tool to inform research on violent deaths, they are subjected to potential biases and challenges relating to data collection and abstraction. Many of these challenges are due to the fragmented nature of the US death investigation system, as acknowledged by the NVDRS itself. Each state implements their own medico-legal procedures (Ruiz et al. 2018; Huguet et al. 2012), which vary by the degree of centralization, credentials and training of death investigation personnel (i.e., medical examiners versus coroners), and levels of funding (Hanzlick 2003). This lack of unified investigation procedures may have important implications for documentation and classifications of violent deaths across states and jurisdictions (Rockett et al. 2018, 2014; Breiding and Wiersema 2006; Dailey et al. 2012).

Effective utility of text narratives entails a need to mitigate challenges in the collection and abstraction of the NVDRS while advocating for continuous improvements of this data source.

While many of the original source documents that inform the NVDRS were not designed for research, the NVDRS narratives have increasingly been used to study a range of violent deaths for prevention and intervention efforts within the last decade (Nazarov et al. 2019). As a foundation for future research, this review provides a comprehensive summary of peer-reviewed studies using NVDRS narratives over the past 20 years, highlights potential challenges of these narratives and how they are addressed in the current literature and provides recommendations on utilizing and improving the information potential of the narratives, with an eye to the application of data science tools.

## Methods

### Search strategies

An informationist (L.N.J.) developed search strategies to identify relevant articles, conference abstracts, and government/agency reports that used NVDRS text narratives (or individual state VDRS narratives). From the time of inception of each database, PubMed, PsycInfo, Scopus, and Google Scholar (for gray literature) were searched on March 26, 2021; updated searches in each database were conducted on January 26, 2022. Each search utilized title and abstract tags for the following keywords and phrases: “National Violent Death Reporting System”, “Violent Death Reporting System”, NVDRS, VDRS, violent, violence, injury, suicide, homicide, “firearm accident”, “unintentional firearm”, “undetermined death”, accident, “intimate partner violence”, IPV, “domestic violence”, “child abuse”, “legal intervention”, “law enforcement”, narrative, “text narrative”, “mixed method”, circumstances, coding, and code. No indexing languages were used since the phrase "National Violent Death Reporting System" is not an indexed term in any of the databases. A set of sentinel articles were identified before the search process to generate search terms and test the effectiveness of the strategies in each database (Barber et al. 2016; Nazarov et al. 2019; Skopp et al. 2019; Ream 2020; Mezuk et al. 2003). The search was not limited by language, publication date, or any other restrictions. Complete search strategies are described in Additional File 1: Appendix A.

### Criteria for study selection

Studies were eligible for full-text abstraction if they were peer-reviewed published articles or government/agency reports in English language that used NVDRS text narratives or individual state VDRS narratives, with no restrictions on the types of study and types of violent death. Two articles that used the NVISS, the predecessor to the NVDRS, were also included. Theses, dissertations, conference presentations and posters, editorials, commentaries, or abstract-only publications were excluded for quality control (Taylor et al. 2014).

### Study selection process

In the first stage, two authors (L.N.D., E.T.K.) independently screened the titles and abstracts of all studies generated from the database search for the following phrases: “National Violent Death Reporting System”, “Violent Death Reporting System”, “NVDRS”, and “VDRS”. Studies were included for further review when the title and abstract screening was inconclusive. Interrater agreement, assessed by comparing screening results of 25 randomly selected articles between two authors, yielded high agreement, with 24 out of 25 articles agreed. Next, the same authors conducted a full-text screening of eligible articles selected from the title/abstract screening to determine whether the text narratives were used in the methods. Any additional articles/reports were identified by screening the references of abstracted articles. Disagreements were resolved through discussions among all authors.

### Data abstraction

The following information was extracted from each article: name of first author, year of publication, type of data (NVDRS, state-specific VDRS, or NVISS), type of death, research question(s), study population(s), study sample size, number of narratives used, type of narratives, selection criteria for narratives, statistical approaches (e.g., purpose for analyzing narratives, methods to analyze narratives, linkage with external data sources), assessment of narrative quality (e.g., efforts to address missing narratives, validation of data abstracted from the narratives), challenges and recommendations pertaining to the narratives and NVDRS as noted by the authors. A description of each extraction variable is provided in Additional File 2: Table S1. Analyses for this study were pre-registered via the Open Science Framework (OSF) in July of 2022 (Johns et al. 2022).

### Summarizing

Frequencies of abstracted articles were described by type of data, type of narratives (C/ME, LE, or both), type of deaths (suicide, homicide, homicide followed by suicide, legal intervention, unintentional firearm, undetermined intent, and multiple types of death), study population (summarized by age groups, gender, professions, health conditions, and vulnerable/minority subgroups), purpose for analyzing narratives, and approaches to assess data completeness and reliability (missing narratives, linkage to external data sources, validation of information abstracted from narratives). In addition, a cumulative flow diagram of studies using the text narratives by methodological tools was generated for the period from 2004 to 2022. Finally, major challenges frequently encountered by researchers, both relating to the narratives and the NVDRS system in general, were summarized.

### Assessment of study quality

The relative quality of studies in terms of sample size, study population, and methodological approaches for analyzing text narratives was evaluated as part of the article abstraction process. However, because we did not seek to derive an overall effect size of a particular exposure-outcome relationship, metrics for assessment of study quality and risk of bias (e.g., Cochrane, Newcastle–Ottawa Scale, etc.) were not relevant for this scoping review (Khalil et al. 2016; Peters et al. 2015).

## Results

### Search results

**Fig. 1:**
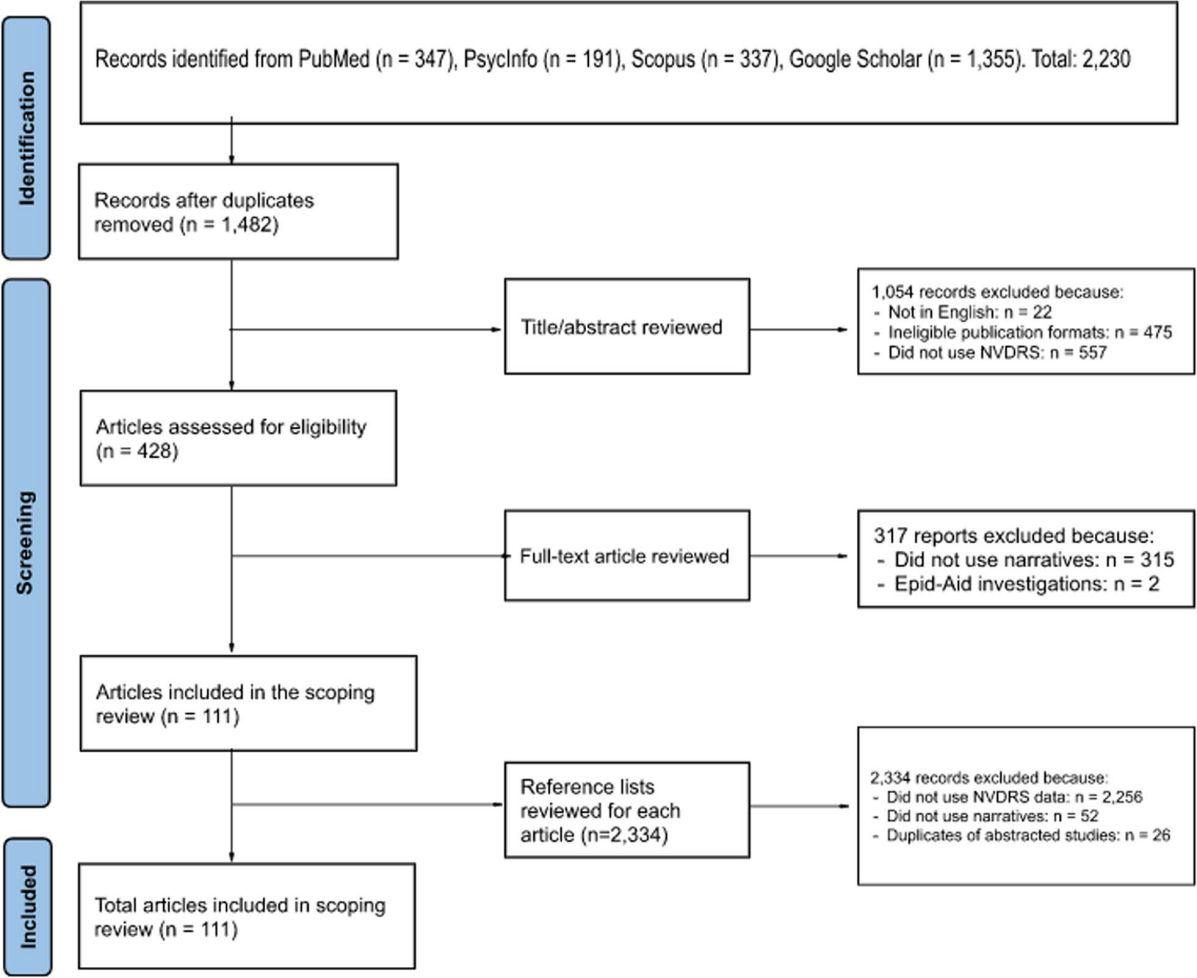
PRISMA Flowchart on Study Identification, Screening, and Inclusion. Source: Page MJ, McKenzie JE, Bossuyt PM, Boutron I, Hoffmann TC, Mulrow CD, et al. The PRISMA 2020 statement: an updated guideline for reporting systematic reviews. BMJ 2021;372:n71. https://doi.org/10.1136/bmj.n71

Figure 1 is a Preferred Reporting Items for Systematic reviews and Meta-Analyses (PRISMA) flow diagram of the study selection process (Page et al. 2020). The initial database search yielded 1820 eligible studies and additional 410 were identified from an updated search (347 in PubMed, 191 in PsycInfo, 337 in Scopus, and 1355 in Google Scholar). After removing duplicates, 1,482 remained for further review. The title/abstract screening identified 428 studies eligible for full-text screening, excluding studies that were not in English (*n* = 22), not peer-reviewed published articles or government/agency reports (*n* = 475), and did not use NVDRS or state VDRS as indicated in the titles and abstracts (*n* = 557). Of the 428 studies, the full-text screening identified 111 eligible for abstraction. No government/agency reports used text narratives and were excluded. Two Epid-Aid reports that used the NVDRS in conjunction with other publicly available data sources as part of the investigations of suicidal behaviors among youth in Utah and Santa Clara Country, California, were excluded (Garcia-Williams et al. 2016; Annor et al. 2017). Finally, the reference screening did not identify any additional studies for inclusion in the full-text abstraction. In summary, a total of 111 studies were included for full-text abstraction. Additional File 3: Table S2, provides descriptions of these studies.

### Characteristics of abstracted studies

As shown by Table 1, more than three quarters of studies (*n* = 91, 82%) used the NVDRS as opposed to state-specific VDRS, and most studies used both C/ME and LE reports (*n* = 106, 95%). Of 111 studies using text narratives, almost half (*n* = 48) studied suicide only; one fifth (*n* = 25) studied homicide (including single, multiple, and mass homicide); and the remaining studied homicide followed by suicide (*n* = 8), legal intervention deaths (*n* = 6), unintentional firearm deaths (*n* = 4), undetermined intent deaths (*n* = 1), and multiple types of deaths (*n* = 16). Many studies were conducted within a particular subpopulation, defined by age groups (19 studies on infants/children, 2 studies on middle-aged adults, and 8 studies on adults aged 50 +); sex/gender or orientation (6 studies on women, 5 studies on men, and 4 studies on LGBTQ +); professions (4 studies on active duty or veterans, 5 studies on healthcare professionals [e.g., nurses, physicians, psychologists], and 3 studies on farmers); health conditions (1 study on cancer, 5 studies on mental/brain disorders, and 1 study on chronic pain), and vulnerable groups (3 studies on pregnant/postpartum women, 1 study on Non-Hispanic Asians/Pacific Islanders, and 6 studies on currently/formerly incarcerated individuals).

**Table 1:** Descriptive characteristics of all included studies and their citations (Peters et al. 2015)

Characteristics of studies	Number of studies	Citation
*Type of data*		
NVDRS	91	Abolarin et al.(2019); Adhia et al.(2019a); Adhia et al.(2020); Adhia et al.(2019b); Arseniev-Koehler et al.(2020); Arseniev-Koehler et al.( 2021); Azrael et al.(2016); Barber et al.( 2016); Barber and Hemenway (2011); Barber et al.( 2021); Blair et al.(2016a); Braun et al.( 2021); Bush(2020); Choi et al.(2019a); Choi et al.(2017); Choi et al.(2019b); Clark et al.(2020); Conner et al.(2019); Craun et al.(2022); Davidson et al.(2021a); Davidson et al.(2021b); De Veauuse Brown and Watson(2022); DeBois et al.(. 2020); DeGue et al.( 2016); Dixon et al.(2020); Fowler et al.(2015); Fowler et al.(2021); Fraga Rizo et al.(2021); Frazier et al.(2017); Gold et al.(2022); Graham et al.(2022); Graham et al.(2021); Hemenway et al.( 2010); Hemenway and Solnick( 2015); Hemenway and Solnick( 2017); Holland et al.(2018); Holland et al.(2017); Hunter et al.(2022); Kafonek et al.(2022); Karch and Nunn(2011); Karch et al.(2013); Kennedy et al.(2021); Klevens and Leeb( 2010); Ko et al.(2021); Logan et al.(2019); Logan et al.(2015); Logan et al.(2013); Logan et al.( 2008); Lohman et al.(2021); Lord( 2012); Lord(2014); Lyons et al.(2019); Lyons et al.(2021a); Lyons et al.(2021b); Massetti et al.(2018); McNally et al.(2016); Mennicke et al.(2021); Mezuk et al.(2003); Mezuk et al.(2021); Michaels and Letson(2021); Miller et al.(2021); Miller and Rensing( 2021); Murfree et al.(2022); O’Donnell et al.(2019); Orlins et al.(2021); Patton et al.(2017); Petrosky et al.(2018); Ream( 2020); Ream(2019); Roberts et al.(2019); Robiner and Li( 2022); Ruch et al.( 2019); Ruch et al.(2021); Schiff et al.(2015); Schmutte et al.(2021); Schwab-Reese et al.(2021); Shawon et al.(2021); Skopp et al.(2019); Smith et al.(2011); Smith et al.(2014); Solnick and Hemenway( 2019); Sordello and Small( 2020); Stone et al.(2016); Tian et al.(2016); Tian et al.(2019); Wallace et al.(2020); Wasserman and Stack( 2011); Wertz et al.(2020); Williams et al.(2018); Wong et al.( 2022); Yau and Paschall( 2018)
State-specific VDRS	18	Annor et al.( 2019); Austin et al.(2016); Briker et al.(2019); Brown and Seals( 2019); Hempstead et al.( 2013); Jiang et al.(2018); Kafka et al.( 2021); Kohlbeck et al.(2020); Kohlbeck et al.(2022); Lavender et al.(2016); Mason et al.( 2021); Mezuk et al.(2015); Presser et al.(2022); Scheyett et al.(2013); Scheyett et al.(2019); Weis et al.(2006); Yousuf et al.(2017); Zeoli et al.(2021)
NVISS	2	Fujiwara et al.(2009); Gabor et al.(2008)
*Type of narratives*		
Coroners/medical examiners	3	Briker et al.( 2019); Ko et al.(2021); Mezuk et al (2019)
Law enforcement	1	Robiner and Li( 2022)
Both	106	Abolarin et al.(2019); Adhia et al.(2019a); Adhia et al.(2019b); Adhia et al.(2020); Annor et al.(2019); Arseniev-Koehler et al.(2020); Arseniev-Koehler et al.(2021); Austin et al.(2016); Azrael et al.(2016); Barber and Hemenway(2011); Barber et al.(2016); Barber et al.(2021); Blair et al.(2016a); Braun et al.(2021); Brown and Seals( 2019); Bush(2020); Choi et al.(2019a); Choi et al.(2017); Choi et al.(2019b); Clark et al.(2020); Conner et al.(2019); Craun et al.(2022); Davidson et al.(2021a); Davidson et al.(2021b); De Veauuse Brown and Watson(2022); DeBois et al.(2020); DeGue et al.(2016); Dixon et al.(2020); Fowler et al.(2015); Fowler et al.(2021); Fraga Rizo et al.(2021); Frazier et al.(2017); Fujiwara et al.(2009); Gabor et al.(2008); Gold et al.(2022); Graham et al.(2022); Graham et al.( 2021); Hemenway et al.( 2010); Hemenway and Solnick( 2015); Hemenway and Solnick( 2017); Hempstead et al.(2013); Holland et al.(2018); Holland et al.(2017); Hunter et al.(2022); Jiang et al.(2018); Kafka et al.(Kafka et al. 2021); Kafonek et al (2022); Karch and Nunn(2011); Karch et al.(2013); Kennedy et al.(2021); Klevens and Leeb( 2010); Kohlbeck et al.(2020); Kohlbeck et al.(2022); Lavender et al.(2016); Logan et al.(2019); Logan et al.(2015); Logan et al.(2013); Logan et al. (2008); Lohman et al.(2021); Lord(2012); Lord( 2014); Lyons et al. (2019); Lyons et al.(2021a); Lyons et al.(2021b); Mason et al.(2021); Massetti et al.(2018); McNally et al.(2016); Mennicke et al.(2021); Mezuk et al.(2015); Mezuk et al.(2021); Michaels and Letson(2021); Miller et al.(2021); Miller and Rensing( 2021); Murfree et al.(2022); O’Donnell et al.(2019); Orlins et al.(2021); Patton et al.(2017); Petrosky et al.(2018); Ream( 2020); Ream(2019); Roberts et al (2019); Ruch et al.(2019); Ruch et al.(2021); Scheyett et al.(2013); Scheyett et al.( 2019); Schiff et al.( 2015); Schmutte et al.(2021); Schwab-Reese et al.( 2021); Shawon et al.(2021); Skopp et al.(2019); Smith et al.(2011); Smith et al.(2014); Solnick and Hemenway( 2019); Sordello and Small( 2020); Stone et al.(2016); Tian et al.(2016); Tian et al.(2019); Wallace et al.(2020); Wasserman and Stack( 2011); Weis et al.(2006); Wertz et al.(2020); Williams et al.(2018); Wong et al.(2022); Yau and Paschall( 2018); Yousuf et al.(2017); Zeoli et al.( 2021)
Unspecified	1	Presser et al.( 2022)
*Type of deaths*		
Suicide	48	Azrael et al.(2016); Barber et al.(2021); Brown and Seals( 2019); Choi et al.(2019a); Choi et al.(2017); Choi et al.(2019b); Clark et al.(2020); Davidson et al.(2021a); Davidson et al.(2021b); Dixon et al.(2020); Fowler et al.(2015); Gold et al.(2022); Hempstead et al.( 2013); Holland et al.( 2017); Karch et al.(2013); Kennedy et al.(2021); Kohlbeck et al.(2020); Kohlbeck et al.(2022); Logan et al (2015); Lohman et al.(2021); Lyons et al.(2019); Mason et al.(2021); Massetti et al.(2018); Mennicke et al.(2021); Mezuk et al.(2003); Mezuk et al.(2015); Miller et al.(2021); O’Donnell et al.(2019); Orlins et al.(2021); Petrosky et al (2018); Ream(2020); Ream( 2019); Roberts et al.( 2019); Ruch et al.(2019); Ruch et al.(2021); Scheyett et al.(2019); Schiff et al.(2015); Schmutte et al.(2021); Skopp et al.(2019); Stone et al.(2016); Tian et al.(2016); Tian et al.(2019); Wasserman and Stack(2011); Weis et al.(2006); Williams et al.(2018); Wong et al.(2022); Yau and Paschall( 2018)
Homicide (single, multiple, and mass homicide)	24	Abolarin et al.(2019); Adhia et al.(2019a); Adhia et al.(2019b); Blair et al.(2016a); De Veauuse Brown and Watson(2022); DeBois et al.( 2020); Fowler et al.(2021); Fraga Rizo et al.(2021); Frazier et al.(2017); Fujiwara et al.(2009); Hemenway and Solnick( 2017); Jiang et al.(2018); Kafonek et al.(2022); Karch and Nunn(2011); Lyons et al.(2021a); Lyons et al.(2021b); Presser et al.(2022); Robiner and Li( 2022); Shawon et al.(2021); Smith et al.(2011); Smith et al.(2014); Wallace et al.(2020); Yousuf et al.(2017); Zeoli et al.(2021)
Homicide followed by suicide	9	Adhia et al.(2020); Holland et al.(2018); Logan et al.(2019); Logan et al.(2013); Logan et al.(2008); McNally et al.(2016); Murfree et al.(2022); Patton et al.(2017); Schwab-Reese et al. (2021)
Legal intervention	7	Arseniev-Koehler et al.( 2021);Barber et al10; Conner et al.(2019); DeGue et al.(2016); Lord(2012); Lord (2014); Wertz et al.(2020)
Unintentional firearm	4	Barber et al.(2011); Hemenway et al.(2010); Hemenway and Solnick( 2015); Solnick and Hemenway( 2019)
Undetermined intent	1	Briker et al.(2019)
Multiple types of deaths	18	Arseniev-Koehler et al.(2020); Austin et al.(2016); Braun et al.(2021); Bush(2020); Craun et al.( 2022); Gabor et al.(2008); Graham et al.(2022); Graham et al.(2021); Hunter et al.(2022); Kafka et al.(2021); Klevens and Leeb( 2010); Ko et al.(2021); Lavender et al.(2016); Mezuk et al.(2021); Michaels and Letson(2021); Miller and Rensing( 2021); Scheyett et al.(2013); Sordello and Small( 2020)
*Age groups*		
Infants or children under 18	19	Adhia et al.(2019a); Briker et al.(2019); Fujiwara et al.(2009); Hemenway and Solnick( 2015); Hemenway and Solnick( 2017); Holland et al.(2018); Holland et al.(2017); Hunter et al.(2022); Karch et al.(2013); Klevens and Leeb( 2010); Kohlbeck et al.(2020); Logan et al.(2013); Lyons et al.(2021a); Michaels and Letson(2021); Murfree et al.(2022); Orlins et al (2021); Presser et al.( 2022); Ruch et al.(2021); Sordello and Small( 2020)
Youth or adolescents (i.e., individuals both younger and older than 18)	9	Adhia et al.( 2019b); Adhia et al.(2020); Bush(2020); Choi et al.(2017); Clark et al.(2020); Graham et al.(2022); Ream(2020); Ream(2019); Ruch et al.(2019)
Young adults (e.g., 18–34 years)	1	O’Donnell et al.(2019)
Middle-aged adults	2	Schiff et al.(2015); Stone et al.(2016)
Older adults (i.e., 50 +)	8	Choi et al.( 2019b); DeBois et al.(2020); Karch and Nunn(2011); Ko et al.(2021); Lohman et al.(2021); Mezuk et al.( 2003); Mezuk et al.(2015); Shawon et al.(2021)
*Gender*		
Men	5	Arseniev-Koehler et al.( 2021); Hempstead et al.(2013); Logan et al.(2019); O’Donnell et al.(2019); Schiff et al.(2015)
Women	6	Austin et al.( 2016); De Veauuse Brown and Watson(2022); Kafonek et al92; Mennicke et al43; Miller and Rensing108; Wallace et al124
LGBTQ +	4	Clark et al.(2020); Lyons et al.(2019); Ream( 2020); Ream(2019)
*Professions*		
Active duty or veterans	4	Logan et al.(2015); O’Donnell et al.(2019); Patton et al.(2017); Skopp et al.(2019)
Healthcare professionals (e.g., nurses, physicians, psychologists)	5	Braun et al.(2021); Davidson et al.(2021a); Davidson et al.(2021b); Gold et al.(2022); Robiner and Li( 2022)
Farmers and agricultural workers	3	Kennedy et al.(2021); Kohlbeck et al.(2022); Scheyett et al.(2019)
*Health conditions*		
Cancer	1	Massetti et al.(2018)
Mental/brain disorders or injury	5	Annor et al.(2019); Miller et al.(2021); Schmutte et al.(2021); Tian et al.(2016); Tian et al(2019)
Chronic pain	1	Petrosky et al.(2018)
*Vulnerable/minority subgroups*		
Pregnant or postpartum women	3	Austin et al.(2016); Miller and Rensing( 2021); Wallace et al.(2020)
Non-Hispanic Asians/Pacific Islanders	1	Wong et al.(2022)
Currently or formerly incarcerated individuals	6	Choi et al.(2019a); Dixon et al.(2020); Fraga Rizo et al.(2021); Mennicke et al.(2021); Ruch et al.(2019); Scheyett et al.(2013)
*Purpose for analyzing narratives*		
Thematic analysis of circumstances of death	38	Adhia et al.( 2019b);Arseniev-Koehler et al.(2020); Arseniev-Koehler et al.(2021); Briker et al.(2019); Brown and Seals( 2019); Choi et al.(2019b); Davidson et al.(2021a); Davidson et al.(2021b); DeBois et al.(2020); Gabor et al.(2008); Holland et al.(2018); Holland et al.(2017); Hunter et al.(2022); Kafonek et al.(2022); Ko et al.(2021); Kohlbeck et al.(2020); Kohlbeck et al.(2022); Lord(2014); Lyons et al.(2021a); Mennicke et al.(2021); Mezuk et al.(2003); Mezuk et al.(2015); Murfree et al.(2022); Orlins et al.(2021); Ream( 2020); Ream(2019); Ruch et al.(2021); Scheyett et al.(2013); Schiff et al.(2015); Schwab-Reese et al.(2021); Shawon et al.(2021); Skopp et al.(2019); Smith et al.(2011); Stone et al.(2016); Wertz et al.(2020); Wong et al.(2022); Yau and Paschall( 2018); Yousuf et al.(2017)
Case identification	49	Abolarin et al.(2019); Adhia et al.(2019a); Adhia et al.(2020); Annor et al.(2019); Austin et al.(2016); Azrael et al.(2016); Barber et al.(2011); Barber et al.(2016); Barber et al.(2021); Blair et al.(2016a); Braun et al.(2021); Bush (2020) Clark et al.(2020); Conner et al.(2019); Craun et al.(2022); DeGue et al (2016); Dixon et al.(2020); Frazier et al.(2017); Gold et al.(2022); Graham et al.(2022); Graham et al.(2021); Hemenway and Solnick( 2015); Jiang et al.(2018); Kafka et al.(2021); Karch et al.(2013); Kennedy et al.(2021); Lavender et al.(2016); Logan et al.(2015); Logan et al.(2008); Lohman et al.(2021); Lyons et al.(2021a); Massetti et al.(2018); McNally et al.(2016); Michaels and Letson(2021); Miller et al.(2021); Miller and Rensing( 2021); O’Donnell et al.(2019); Ream( 2019); Roberts et al.(2019); Robiner and Li( 2022); Schmutte et al.(2021); Sordello and Small( 2020); Tian et al.(2016); Tian et al.(2019); Wallace et al.(2020); Wasserman and Stack( 2011); Weis et al.(2006); Williams et al.(2018); Zeoli et al.(2021)
Both thematic analysis and case identification	23	Choi et al.( 2019a); Choi et al.(2017); De Veauuse Brown and Watson(2022); Fowler et al.(2015); Fowler et al.( 2021); Fraga Rizo et al.(2021); Fujiwara et al.(2009); Hemenway et al.( 2010); Hemenway and Solnick( 2017); Hempstead et al.( 2013); Karch and Nunn (2011); Klevens and Leeb( 2010); Logan et al.(2019); Logan et al.(2013); Lord(2012); Lyons et al.(2021b); Mason et al (2021); Patton et al.(2017); Petrosky et al.(2018); Presser et al.(2022); Scheyett et al.(2019); Smith et al.(2014); Solnick and Hemenway( 2019)
Evaluate missingness and length of narratives	1	Mezuk et al.(2021)
*Methodological tools for analyzing narratives*		
Manual review	81	Abolarin et al.(2019); Adhia et al.(2019a); Adhia et al.( 2020); Adhia et al.(2019b); Barber and Hemenway(2011); Barber et al (2016); Barber et al.(2021); Blair et al.(2016a); Braun et al.(2021); Briker et al (2019); Brown and Seals( 2019); Bush(2020); Choi et al.( 2019a); Choi et al.(2017); Conner et al.( 2019); Davidson et al.(2021b); DeBois et al.(2020); DeGue et al.( 2016); Dixon et al.(2020); Fowler et al.(2021); Fraga Rizo et al.(2021); Frazier et al (2017); Fujiwara et al (2009); Gabor et al.(2008); Gold et al.(2022); Graham et al (2022); Graham et al.(2021); Hemenway et al.(2010); Hemenway and Solnick( 2015); Hemenway and Solnick( 2017); Hempstead et al.( 2013); Holland et al.(2018); Holland et al.( 2017); Hunter et al.(2022); Jiang et al.(2018); Kafka et al.( 2021); Kafonek et al.(2022); Karch and Nunn(2011); Karch et al.(2013); Kennedy et al (2021); Klevens and Leeb( 2010); Kohlbeck et al.(2020); Kohlbeck et al.(2022); Lavender et al(2016); Logan et al.(2019); Logan et al.(2019); Logan et al.( 2013); Logan et al.(2008); Lord( 2012); Lord(2014); Mason et al (2021); McNally et al.( 2016); Mennicke et al.(2021); Mezuk et al.(2015); Michaels and Letson(2021); Miller and Rensing( 2021); Murfree et al.(2022); Orlins et al.( 2021); Patton et al.(2017); Presser et al.(2022); Ream(2020); Robiner and Li (2022); Ruch et al.( 2019); Ruch et al.(2021); Scheyett et al.(2013); Scheyett et al.(2019); Schiff et al.(2015); Schmutte et al.(2021); Schwab-Reese et al.( 2021); Shawon et al.(2021); Skopp et al.(2019); Smith et al.(2011); Smith et al.(2014); Solnick and Hemenway( 2019); Sordello and Small( 2020); Stone et al.(2016); Weis et al.(2006); Wertz et al.(2020); Williams et al.(2018); Wong et al.(2022); Yousuf et al.(2017); Zeoli et al.( 2021)
Keyword search	9	Annor et al.( 2019); Clark et al.(2020); Fowler et al.(2015); Lyons et al.( 2019); Massetti et al.(2018); Petrosky et al.(2018); Ream( 2019); Schmutte et al.(2021); Yau and Paschall(2018)
Both keyword search and manual review	13	Azrael et al.(2016); Choi et al.(2019b); Craun et al.(2022); De Veauuse Brown and Watson(2022); Lyons et al.(2021a); Lyons et al.( 2021b); Miller et al.(2021); O’Donnell et al.(2019); Roberts et al.( 2019); Tian et al (2016); Tian et al. (2019); Wallace et al.(2020); Wasserman and Stack( 2011)
Natural language processing or data science	6	Arseniev-Koehler et al.( 2020); Arseniev-Koehler et al.( 2021); Davidson et al.(2021a); Ko et al.(2021); Lohman et al.(2021); Mezuk et al.(2003)
Other/unclear	2	Austin et al.( 2016); Mezuk et al.(2021)
*Assessment of data completeness and reliability*		
Address missing narratives	17	Adhia et al.( 2019a); Barber et a (2016); Brown and Seals( 2019); Choi et al.(2017); Davidson et al.(2021a); Gabor et al.(2008); Kafonek et al.(2022); Klevens and Leeb( 2010); McNally et al.(2016); Mezuk et al.(2021); Mezuk et al.(2003); Scheyett et al.(2019); Shawon et al.(2021); Skopp et al.(2019); Wertz et al.( 2020); Wong et al.(2022); Yau and Paschall( 2018)
Linkage to external data sources	36	Annor et al.( 2019); Arseniev-Koehler et al.( 2021); Austin et al.(2016); Barber and Hemenway(2011); Barber et al.(2016); Conner et al.( 2019); DeBois et al.(2020); Fowler et al.( 2021); Gold et al.(2022); Graham et al.(2022); Graham et al.(2021); Hemenway and Solnick( 2015); Hemenway and Solnick( 2017); Hempstead et al.( 2013); Ko et al.(2021); Lavender et al (2016); Logan et al.(2015); Lohman et al.(2021); Mezuk et al.( 2015); O’Donnell et al.(2019); Petrosky et al.(2018); Ream (2020); Ream(2019); Robiner and Li( 2022); Scheyett et al.(2013); Shawon et al.(2021); Skopp et al.(2019); Solnick and Hemenway( 2019); Tian et al.(2016); Tian et al.(2019); Wallace et al (2020); Weis et al.(2006); Wertz et al.(2020); Williams et al.(2018); Yau and Paschall (2018); Zeoli et al.(2021)
Assess agreement between narratives and quantitative coded variables	48	Adhia et al.( 2019a); Arseniev-Koehler et al.(2021); Azrael et al.(2016); Barber and Hemenway(2011); Barber et al (2016); Braun et al.(2021); Brown and Seals (2019); Bush(2020); Choi et al.(2019a); Conner et al.(2019); Davidson et al57; Davidson et al.(2021b); DeGue et al.(2016); Fowler et al.( 2015); Fowler et al.(2021); Fraga Rizo et al.( 2021); Frazier et al.(2017); Fujiwara et al.(2009); Gold et al.(2022); Graham et al.(2022); Graham et al.(2021); Hemenway et al.( 2010); Hemenway and Solnick( 2015); Hemenway and Solnick( 2017); Kafka et al.(2021); Karch and Nunn(2011); Karch et al.(2013); Klevens and Leeb( 2010); Lavender et al.( 2016); Logan et al.(2019); McNally et al.(2016); Mennicke et al.(2021); Mezuk et al.(2003); O’Donnell et al.(2019); Orlins et al.(2021); Patton et al.( 2017); Ream(2020); Ream(2019); Ruch et al.(2021); Schiff et al.( 2015); Smith et al.(2011); Solnick and Hemenway(2019); Sordello and Small( 2020); Stone et al.(2016); Wallace et al.( al. 2020); Weis et al.(2006); Wertz et al.(2020); Yau and Paschall(2018)
Assess agreement between narratives and external data	6	Barber et al.(2011); Barber et al.(2016); Conner et al.(2019); Klevens and Leeb(2010); Robiner and Li (2022); Williams et al.(2018)
Assess agreement between C/ME and LE narratives	1	Gabor et al. (2008)

### Assessment of data completeness and reliability

Only a few studies reported missing narrative data (*n* = 17), and the majority failed to specify whether missing narrative data were of significant concern to the research question(s), how and/or why a particular narrative was missing, as well as how missingness was handled. Almost half of studies (*n* = 48) assessed the degree to which similar information agreed between the quantitative coded variables and qualitative text narratives. One-third (*n* = 36) used or linked to external data sources beyond the NVDRS or state VDRS, for example, the US Census data (for mortality data), medical records (for additional health characteristics), and media reports (for additional case identification). Out of 36 studies that linked to external data sources, the majority (*n* = 30, 83%) did not assess the degree to which similar information agreed between the narratives and/or NVDRS variables with the external sources (Table 1).

### Purpose for analyzing narratives

Narratives were used in two distinct ways. The majority of studies analyzed contents of narratives to characterize salient risk factors or circumstances around deaths (*n* = 38, 34%) or to supplement existing quantitative variables for case identification (*n* = 49, 44%), or both (*n* = 23, 21%) (Table 1). For example, Adhia et al. (2020) manually reviewed text narratives to characterize murder-suicides perpetrated by adolescents. Arseniev-Koehler et al. (2021) employed a topic modeling approach to investigate racial and ethnic differences in the narrative descriptions of threat and dangerousness (e.g., physical aggression) associated with legal intervention deaths among men.

### Methodological tools for analyzing narratives

There were a wide range of statistical approaches used for analyzing the narratives. As shown in Table 1, narratives were primarily analyzed through manual review (*n* = 81, 73%), keyword searches (*n* = 9, 8%), or a combination of approaches (*n* = 13, 12%). Only a few studies employed data science methods including natural language processing (*n* = 3) and topic modeling (*n* = 3). (Adhia et al. 2020; Arseniev-Koehler et al. 2021).

**Fig. 2:**
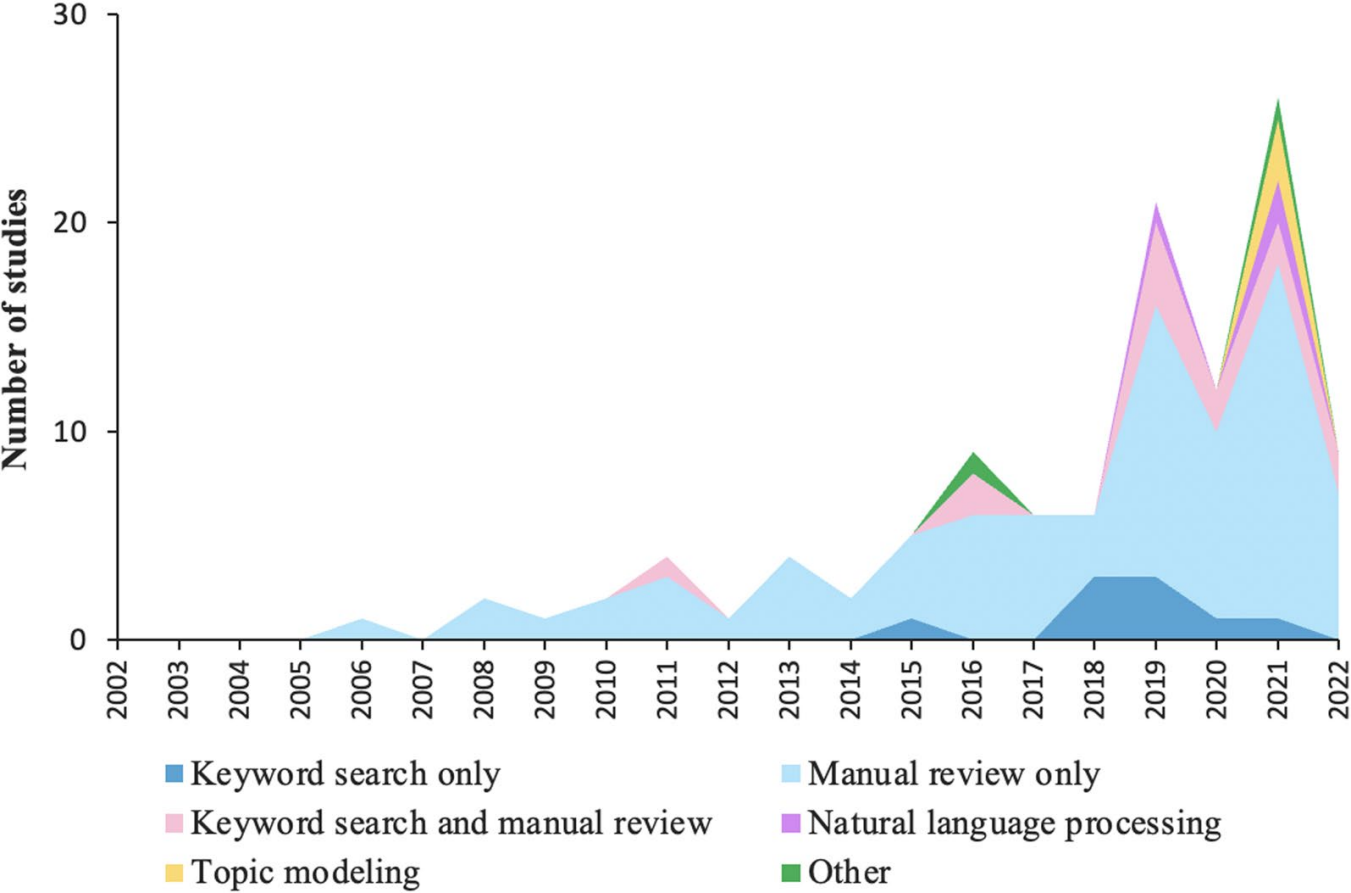
Cumulative flow diagram of studies using text narratives by methodological tools between 2002 and 2022

Figure 2 shows the cumulative flow diagram of studies using the text narratives by methodological tools in the period between 2002 and 2022, as the NVDRS began collecting data in 2002 (Center and for Injury Prevention and Control, Division of Violence Prevention 2021). Studies that used text narratives were first published four years after the creation of NVDRS; the number of these studies increased over time, with the overwhelming majority being published after 2014 (*n* = 94, 85%). Notably, there was a shift in the methodological tools used for analyzing the narratives over time. Methods for analyzing narratives became increasingly diverse; for example, there were a growing number of studies employing keyword search, natural language processing, and topic modeling in addition to manual review in recent years. Additionally, more advanced statistical methods were used to extract narrative data. While manual review was predominantly and exclusively used in studies prior to 2015, more studies have used keyword search since 2015 and data science methods (e.g., natural language processing and topic modeling) since 2019.

### Data challenges encountered by researchers

Table 2 summarizes two major challenges frequently encountered by the researchers. The first challenge relates to a lack of or limited information on contextual factors relevant to deaths or populations being investigated. For example, several studies found that demographic and circumstantial details in the narratives were insufficient for case identification and characterization of death incidents. (Scheyett et al. 2013; Frazier et al. 2017; Briker et al. 2019; Fraga Rizo et al. 2021) Sensitive topics such as child maltreatment, intimate partner homicides, and legal intervention deaths, while routinely collected by the NVDRS, are limited to the information provided by the source documents and interpretations of the abstractors. (Lord 2014; Brown and Seals 2019; Hunter et al. 2022) The second challenge relates to information variation within the NVDRS system, such as discrepancies between different data sources (e.g., C/ME and LE reports) and variations in reporting, coding, abstraction, completeness, and contents of text narratives and NVDRS across states.

**Table 2:** Major challenges encountered by researchers relating to the text narratives and NVDRS system in general

	Challenges relating to text narratives	Challenges relating to NVDRS/state VDRS data
Challenge 1: *Lack of or limited information on contextual factors relevant to deaths or populations being investigated*	Limited or insufficient details on relevant demographic and circumstantial data in the narratives for case identification and characterization of deaths Blank or uninformative narratives (e.g., contents have little or no descriptions of circumstances around death)	Certain demographic and circumstance variables are not routinely collected or readily available for the researchers (e.g., medical records, data on child abuse) Missingness in certain demographic and circumstance variables (e.g., mental health history) Missing or incomplete data due to ongoing investigations or deaths occurring in states different from state of residence
Challenge 2: *Variation in reporting, coding and abstraction, and narrative information potential*	Narratives are collected from informants and third parties (e.g., family/friends of the decedents) and limited to information known by coroner/medical examiner and law enforcement Contents of narratives depend on the interpretations and/or information deemed relevant by the abstractors Human errors in coding/abstraction process Level of details, missingness, and conflicting information from coroner/medical examiner and law enforcement narratives Variations in length, depth, completeness, and availability across county/state, characteristics of the decedent, and types of death could limit data-sharing	Differences in data collection, availability, coding and abstraction procedures across counties/states Variation in the degree of missingness depending on the nature of the data (e.g., toxicology and sensitive topics such as intimate partner violence are frequently missing) Inconsistencies between different data sources (e.g., data collected from family, friends, coroners/medical examiners, and law enforcement) Inconsistencies between the narratives and coded variables

### Quality of included studies

All studies included in this scoping review were peer-reviewed, which serves as a crude metric of research quality. The sample size of included studies (ranged from 46 to 233,108 incidents) was appropriate for the research questions, which were largely descriptive and representative of the decedents in the population of interest. Most studies limited their sample to cases from continuously reporting NVDRS states to ensure the reliability of narrative data. Whether they used traditional qualitative techniques or data science tools, studies employed rigorous methodological approaches for analyzing narratives. For example, many studies (e.g., Holland et al. (2017) Kohlbeck et al. (2020) Schwab-Reese et al. (2021) Mennicke et al. (2021)) developed comprehensive coding guidelines for characterizing salient circumstances of violent deaths via open-coding procedures and comparative methods. Other studies (e.g., Tian et al. (2016) Petrosky et al. (2018) O’Donnell et al. (2019) Miller et al. (2021)) improved case identification by employing keyword searches followed by manual review of the narratives.

## Discussion

This review provides a comprehensive assessment of the research utility of the NVDRS text narratives as a valuable qualitative tool for understanding violence at the population scale. Results showed a substantial increase in the number of studies using the narrative data in recent years, particularly concerning correlates of suicide and homicide consistent with prior reviews of the NVDRS (Nazarov et al. 2019). Leveraging text narratives in studying suicide deaths presents a unique opportunity for identifying novel risk factors and advancing the historically stagnant nature of suicide research (Franklin et al. 2017). This review also highlights that taking full advantage of NVDRS narratives will require novel methodological tools, including those captured under the umbrella of “data science”, to extract insights from these narratives in an effective and meaningful way. These tools, in turn, will be enhanced by integrating and incorporating multiple data sources to understand both protective and risk factors to go beyond the purely descriptive nature of many of the studies included here.

This review identified several data challenges that researchers have frequently encountered; many of which align with previously identified limitations of the RAD-NVDRS (Kaplan et al. 2017). First, relevant contextual factors are often lacking or insufficient in the narratives. The NVDRS, and its narrative data, depend on the completeness and accuracy of the original C/ME and LE sources; both of which are dependent on the nature of violent deaths, death investigation procedures, qualifications, and experiences of the data abstractors, as well as the relationships between various local and state level stakeholders. For example, toxicological reports and sensitive information, such as circumstances around child maltreatment, intimate partner homicides, and legal intervention deaths, are often missing. Further, detailed contextual information around relationship status (Abolarin et al. 2019; Smith et al. 2014), the presence of cyber abuse and bullying (Brown and Seals 2019), and diagnosed mental health and substance use (Mezuk et al. 2015; Logan et al. 2008) were identified as lacking or insufficient.

Additionally, many studies reported the difficulties of capturing relevant circumstantial information due to ongoing investigations, deaths occurring in states different from state of residence, and deaths involving law enforcement suspects. Therefore, any efforts to draw inferences from the narratives require a careful consideration of sources of missingness, both in abstractor-coded variables and text narratives, particularly in studying legal intervention deaths given officers are both the inflictors and key witnesses. Such a dynamic can have implications for the accuracy and presence of important circumstances in the narrative data. This further illustrates how the research question may affect both the awareness and nature of the challenges associated with using narratives.

Second, the review highlighted the challenges relating to variability of the narratives in terms of length, completeness, and availability. As narratives are collected from secondary sources such as suicide notes and interviews with family/friends of the decedents, their contents vary depending on the information reported by the informants, circumstance details deemed relevant by the coroner/medical examiner and law enforcement, as well as the interpretations of the abstractors. These narrative variations may also stem from human errors during coding and abstraction process (Dailey et al. 2012). Information bias can arise when the data presence or quality of narratives varies systematically as a function of decedent characteristics (Mezuk et al. 2021), which has broad implications on the ability to draw unbiased inferences from this data source. These challenges with death certificate data have been previously documented (Data and Surveillance Task 2014).

Third, there are information inconsistencies between various data sources, including conflicting information between C/ME and LE narratives and between the abstractor-coded variables and the narrative texts. These inconsistencies arise because the NVDRS data, while designed as a research repository, are derived from source documents collected for non-research purposes. A lack of or an underdeveloped data-sharing between different partners (e.g., vital records, C/ME offices, law enforcement) can result in inconsistencies within the NVDRS. While the CDC provides detailed Users’ Manuals for the NVDRS (Centers for Disease Control and Prevention 2020, 2021, 2022b), there is a general lack of concrete guidance on how to reconcile incongruencies and integrate text narratives with the abstractor-coded variables. This review found that researchers who utilize the narratives as a means of case *finding* or case *confirming* often privilege the content within the qualitative data in classifying or categorizing cases and incident circumstances when coded variables were found to be insufficient (Davidson et al. 2021a; Lohman et al. 2021; Yau and Paschall 2018; Wertz et al. 2020). However, few studies reported information on missingness or incompleteness of these texts, much less how such data issues were addressed in the analysis.

Lastly, although the NVDRS has expanded to all 50 US states, Puerto Rico, and the District of Columbia, states participate in this reporting system at various points in time. Early participating states (e.g., Virginia, New Jersey) have more established death investigation infrastructures and therefore, more consistent data in comparison with newer states (e.g., California) (Center and for Injury Prevention and Control, Division of Violence Prevention 2021). This can have an impact on the information potential of the narratives. Furthermore, not all states participate in optional modules such as the IPV and CFR modules (Centers for Disease Control and Prevention 2022b). These data barriers may result in small analytical samples, as studies often limited their analyses to states that have consistently reported data.

Informed by the findings from this review, Table 3 summarizes recommendations for improving the utility of text narratives, both for end-users (i.e., researchers) and for NVDRS administrators. Our findings suggest several opportunities for researchers to leverage existing, advanced, and flexible data science methods to explore and analyze large amounts of unstructured textual data in a meaningful and efficient manner. Contrary to traditional textual analysis methods (e.g., manual review, keyword searches), which are often time-consuming and labor-intensive, natural language processing and topic modeling can be immensely useful in combing through large amounts of textual data, detecting patterns in circumstances, and building algorithms as an alternative for manual review, as illustrated by some of the studies included in this review (Mezuk et al. 2003; Lohman et al. 2021; Arseniev-Koehler et al. 2020). However, these data science methods can be computationally intensive, require specialized and technical knowledge, and often rely on the *amount* of data included in narratives which, in turn, rely on the consistent and detailed abstraction of circumstances around violent deaths.

**Table 3:** Recommended approaches to address challenges in utility of text narratives for research and practice

Recommended approaches by NVDRS data users	Strategies
Approach 1: *Employ data science methods for large textual data to improve case identification and characterization of contexts around deaths*	Use topic modeling to characterize salient risk factors and circumstances around violent deaths Use natural language processing algorithms in addition to traditional textual analysis methods to optimize case identification using narratives, particularly for rare and emergent risk factors/outcomes Broadly employ algorithm-based data science tools as an alternative to less efficient methods such as manual review to increase sample size and improve reliability of inferences
Approach 2: *Leverage new and existing linkages to data sources beyond NVDRS*	Link to data sources beyond NVDRS to obtain additional circumstances and risk factors (e.g., population characteristics from US Census and health history from medical records) Supplement case finding with other publicly available data sources, including media reports (e.g., The Washington Post database for fatal police shootings), Vital Statistics, and other violent death databases (e.g., Federal Bureau of Investigation's Uniform Crime Reporting Program) Construct comparison groups matched to NVDRS/VDRS decedents from large population-based surveys, such as the National Survey on Drug Use and Health, National Health Interview Survey, and Youth Risk Behavior Surveillance System, to strengthen the ability to make inferences about risk and protective factors
Approach 3: *Address inconsistencies and improve data integration within the NVDRS*	Provide concrete guidance to address discrepancies between coroners/medical examiners and law enforcement narratives, as well as between abstractor-coded variables and text narratives Identify and investigate how discrepancies across different sources (death certificates, coroners/medical examiners reports, law enforcement reports) arise Strengthen state and federal death investigation systems to improve the quality of the NVDRS Improve transparency in data quality assurance procedures and results
Approach 4: *Engage in dialogue with NVDRS creators and establish best practices for maximizing the research potential of text narratives*	Conduct focus groups with users and creators of the NVDRS/VDRS narratives to identify opportunities for data retrieval and sharing that maximizes the research usefulness of the NVDRS narratives Encourage meaningful collaboration among NVDRS/VDRS data users as well as between data users and creators Facilitate partnerships/collaborations between stakeholders across states and jurisdictions on death investigations, data collection, and abstraction Transparent documentation of data completeness of the text narratives (e.g., report missingness of narratives in published studies, examine systematic differences in length and content of narratives by characteristics of decedents, etc.)

To generate a meaningful comparison group, the NVDRS can be linked to external datasets using temporal (e.g., year) and geographic (e.g., state) identifiers to characterize additional circumstances or contexts (e.g., health circumstances, rurality/urbanicity, etc.), create comparison groups to make inferences about potential risk and protective factors, and for more complete case ascertainment using other sources of violent death reporting. Examples of publicly-available data sources beyond the NVDRS include the Census (Petrosky et al. 2018; Yau and Paschall 2018Graham et al. 2022), other mortality registries and vital records (Barber et al. 2016; Austin et al. 2016), media reports (DeBois et al. 2020; Robiner and Li 2022), and population-based surveys (Hemenway and Solnick 2015), However, data linkage can be difficult given the requirement of identifiers with which to link, the dynamic nature of some data including EMRs, concerns over privacy, and the necessity of “comparable” groups when using non-deceased controls.

Lastly, given a large share of studies utilized the text narratives as a means of supplementing information provided in the coded variables, incongruencies between the narrative and coded variables (or potentially between C/ME versus LE narratives themselves) are an important challenge faced by researchers and, to our knowledge, there is no existing guidance on how to integrate these two data sources, reconcile discrepancies, or when to privilege one over the other. As such, greater transparency and clearer documentation from NVDRS administrators to the research community are needed. A few studies have focused on recommendations for the improvement of the NVDRS, including the standardization of the investigation system and data collection procedures (Kaplan et al. 2017; Friday 2006), although such standardization efforts are challenging due to systemic barriers in infrastructure, limited resources, and funding.

### Strengths and limitations

To the best of our knowledge, this review is the first comprehensive evaluation of the utility of the NVDRS narratives as a valuable qualitative source in studying violent deaths, with a focus on the analytical tools and data challenges with analyzing narrative texts. The restriction to peer-reviewed studies, the relatively large size and representative nature of the sample of eligible studies, well-defined study populations, and various rigorous methodological approaches of the studies reviewed indicates that studies using these narratives are of sufficient quality to draw reliable inferences. A broad range of study populations, exposure-outcome relationships, and research questions were examined, which collectively can inform future research using this data system. This review additionally recommended actionable approaches to enhance the research usefulness of the narratives and NVDRS data. Despite the comprehensive nature of this review, there are several limitations. First, a defined set of major databases were used to capture the scholarly and academic literature at the cost of others (Web of Science, OVID, Embase). Secondly, studies included in this review were limited to peer-reviewed sources and do not include dissertations, posters, abstracts, letters to the editor, and conference proceedings. As a result, findings are subject to publication bias, which can have implications for the resulting conclusions.

## Conclusion

By producing actionable insights and recommendations, this review endeavors to improve and maximize the use of text narratives and NVDRS data in research. Increasing use of advanced data science methods, leveraging linkages to external datasets, and increasing awareness of and addressing issues of narrative completeness and quality are important considerations. By providing guidance on the use of narrative texts, this review furthers the goal of the NVDRS to assess and understand the scope of violent deaths to inform prevention efforts more completely.

## Supplementary Information

**Additional file 1. Appendix A**. Complete Search Strategies This file contains description of the full search strategies conducted for this review.

**Additional file 2. Table l**: Data Extraction Table. This table contains description of each variable extracted from articles included for full-text abstraction in the review.

**Additional file 3. Table 2**: Studies using National Violent Death Reporting SystemText Narratives by Year of Publication, 2002-2022. This table contains descriptive data of all articles included for full-text abstractionin the review.

## Data Availability

The NVDRS is publicly available to researchers and public health practitioners at https://www.cdc.gov/violenceprevention/datasources/nvdrs/dataaccess.html.

## Abbreviations

NVISS: National Violent Injury Statistics System
NVDRS: National Violent Death Reporting System
VDRS: Violent Death Reporting System
CDC: Centers for Disease Control
C/ME: Coroner and medical examiner
LE: Law enforcement
IPV: Intimate partner violence
CFR: Child fatality review
RAD: Restricted access database
EMRs: Electronic medical records
PRISMA: Preferred Reporting Items for Systematic reviews and Meta-Analyses
OSF: Open Science Framework
