# Supplementary material for: Research utility and limitations of textual data in the National Violent Death Reporting System: a scoping review and recommendations
Source: Inj Epidemiol. 2023 May 9;10:23. doi: 10.1186/s40621-023-00433-w (PMC10170777; doi:10.1186/s40621-023-00433-w)
Supplement: Supplementary file 1 — Additional file 1. Appendix A. Complete Search Strategies This file contains description of the full search strategies conducted for this review. [file 40621_2023_433_MOESM1_ESM.docx]

**Appendix A.** Complete Search Strategies

**PubMed:**

"Violent Death Reporting System"[Title/Abstract] OR "NVDRS"[title/abstract]

Results: 291

Ran on 03/26/2021

Updated Search:

Update: "Violent Death Reporting System"[Title/Abstract] OR "NVDRS"[title/abstract] AND (2021/3/26:2022/1/26[pdat])

Results: 56

Ran on 01/26/2022

**PsycInfo:**

TI "Violent Death Reporting System" OR AB "Violent Death Reporting System"

TI NVDRS OR AB NVDRS

(S1 OR S2)

Results: 173

Ran on 03/26/2021

Updated Search:

TI "Violent Death Reporting System" OR AB "Violent Death Reporting System"

TI NVDRS OR AB NVDRS

(S1 OR S2)

Limiters - Published Date: 20210301-20220231

Results: 18

Ran on 01/26/2022

**Google Scholar:**

("Violent Death Reporting System" OR NVDRS OR "national violent death reporting system") AND Narrative

Results: 1060

Ran on 03/26/2021

Search was ran in Publish or Perish by dividing search by 2 dates:

1999-2012

2012-2021

Updated Search on 01/26/2022

Results: 295

**Scopus:**

TITLE-ABS("Violent Death Reporting System") OR TITLE-ABS("NVDRS")

Results: 296

Ran on 03/26/2021

Updated Search:

( TITLE-ABS ( "Violent Death Reporting System" ) OR TITLE-ABS ( "vdr" ) ) AND ( PUBDATETXT ( "March 2021" OR "April 2021" OR "May 2021" OR "June 2021" OR "July 2021" OR "August 2021" OR "September 2021" OR "October 2021" OR "November 2021" OR "December 2021" OR "January 2022" ) )

Results: 41

Ran on 01/26/2022
