# Supplementary material for: Research utility and limitations of textual data in the National Violent Death Reporting System: a scoping review and recommendations
Source: Inj Epidemiol. 2023 May 9;10:23. doi: 10.1186/s40621-023-00433-w (PMC10170777; doi:10.1186/s40621-023-00433-w)
Supplement: Supplementary file 2 — Additional file 2. Table l: Data Extraction Table. This table contains description of each variable extracted from articles included for full-text abstraction in the review. [file 40621_2023_433_MOESM2_ESM.docx]

**Supplemental Table 1.** Data Extraction Table*

| **Data Extraction Variable** | **Description** |
| --- | --- |
| Article | Title of article or report. |
| First Author, Year | The last name of the first author and the year of publication. |
| Timespan for Data Used | The time period of the data used in the study. |
| Exposure | Description of the primary exposure of interest, if applicable. If no exposure is explicitly studied and the study is descriptive, a more detailed description of the type of death could be included. |
| Type of Death(s) | The type of violent death(s) that was studied. Options include: suicide, homicide, homicide followed by suicide, firearm-related deaths, unintentional deaths, legal intervention, etc. |
| Type of Data | Whether the NVDRS, state specific VDRS, or NVISS was used. |
| Research Aim(s) | A description of the research question(s) or aim(s). |
| Study Population | A description of the inclusion and exclusion criteria used in the study to define the population being studied. |
| Sample Size | The number of incident deaths/victims that made up the analytic sample or study population. |
| Number of Narratives | The number of narratives used and analyzed in the study. If not reported, listed as “No”. |
| Criteria for Narrative Selection | The criteria used by authors to select narratives for use/analysis. If all narratives from the study population were used, that could be specified. |
| Type of Narratives | Whether medical examiner narratives, law enforcement narratives, or both were used in the study. Could also list if unspecified in the study. |
| Method for Identifying Narratives | Description of the method used in the study to identify eligible narratives. Options include: manual review, keyword search, natural language processing, unclear, etc. More than one method could also be specified. |
| Linkage with External Data | Whether the study used external data sources or linked NVDRS data with external data sources. If “Yes”, a description is provided. Otherwise “No” is listed. |
| Efforts to Compare | Whether efforts were made in the study to compare information present or abstracted from the narratives with either external data sources or with coded variables within the NVDRS. If “Yes”, a description is provided. Otherwise “No” is listed. |
| Analysis Method(s) | Description of the summary measure(s) and approaches used to analyze content from the narratives. Options include: content analysis, as a supplement to coded variables, data science, regression, etc. More than one method could also be specified. |
| Statistical Approach | Description of the approach used, whether related to narratives or not. Options include: descriptive epidemiology, risk factor analysis, evaluation research, trend analysis, data quality assessment, etc. More than one method could also be specified. |
| Missing Narratives | Whether missing narratives were reported. If “Yes”, a description of the extent of the missingness and how it was handled is provided. Otherwise “No” is listed. |
| Limitations of the Narratives | Description of the limitations of the narratives as provided by the authors of the article or report. If no limitations specific to the narrative are discussed, “N/A” is listed. |
| Other Limitations of the NVDRS | Description of the limitations of the NVDRS more generally as provided by the authors of the article or report. These may also overlap with limitations relating to the narratives. If no limitations specific to the NVDRS more broadly are discussed, “N/A” is listed. |
| Recommendations for Future Research | Description of recommendations for future research made by authors of the study as it relates to the narratives or the NVDRS in general. |
| Other Notes | Description (optional) of other relevant details related to the nature of the article or report (e.g., study is a research letter, a duplicate, or presented at a conference, etc.) |

**All data extraction fields were free text. Response options are provided, when applicable.*
