# Supplementary material for: Research utility and limitations of textual data in the National Violent Death Reporting System: a scoping review and recommendations
Source: Inj Epidemiol. 2023 May 9;10:23. doi: 10.1186/s40621-023-00433-w (PMC10170777; doi:10.1186/s40621-023-00433-w)
Supplement: Supplementary file 3 — Additional file 3. Table 2: Studies using National Violent Death Reporting SystemText Narratives by Year of Publication, 2002-2022. This table contains descriptive data of all articles included for full-text abstractionin the review. [file 40621_2023_433_MOESM3_ESM.docx]

**Supplemental Table 2.** Studies using National Violent Death Reporting System (NVDRS) Text Narratives by Year of Publication, 2002-2022*

| **Article** | **First Author, Year** | **Time period used** | **Type of death** | **Research Aim(s)** | **Study population** | **Sample size** | **Narrative analysis method** | **Linkage with external data** | **Challenges pertaining to the narratives and NVDRS/VDRS*** |
| --- | --- | --- | --- | --- | --- | --- | --- | --- | --- |
| An exploration of human services system contacts prior to suicide in South Carolina: An expansion of the South Carolina Violent Death Reporting System | Weis, 2006 | 2004 | Suicide | To link the South Carolina VDRS with other databases for expanded analysis of suicide deaths | All South Carolina residents | 491  incidents | Manual review | Linked to hospitalization, emergency room visits, diagnoses, and services from South Carolina Department of Mental Health and criminal justice data | None mentioned |
| Characteristics of perpetrators in homicide-followed-by-suicide incidents: National Violent Death Reporting System - 17 US States, 2003-2005 | Logan, 2008 | 2003-2005 | Homicide-suicide | To describe perpetrators of homicide- suicides and compare homicide-suicide perpetrators with suicide-only and homicide-only suspects | Decedents of homicides followed by suicides where perpetrator was known and not multiple perpetrators | 408 incidents | Manual review | No | Narratives were collected from friends/family and limited to information known by medical examiners and LE; information on mental health and substance abuse was limited to the information provided by the source documents and interpretations of the abstractors |
| A comparison of law enforcement and medical examiner reports in a violent-death surveillance system | Gabor, 2008 | 2002-2003 | Homicide and homicide- suicide | To characterize similarities and differences between LE and C/ME reports; to research on the different aspects of implementation of mortality surveillance and their impacts | Decedents of homicide or homicide followed by suicide incidents in Utah that had both C/ME and LE reports | 47 homicides  11 homicide- suicides | Manual review | No | Errors in coding/abstraction; narratives were dependent on the interpretations and/or information deemed relevant by the abstractors; inconsistencies in case definition; variability in data reporting between urban and rural areas and across states |
| Characteristics of infant homicides: findings from a US multi site reporting system | Fujiwara, 2009 | 2001-2002 | Homicide | To describe and characterize subtypes of infant homicides, weapons, and perpetrators involved | Children under age 2 who died by homicides, excluding unintentional vehicular homicides and unintentional deaths involving a caretaker's negligence | 71 incidents | Manual review | No | None mentioned |
| Unintentional firearm deaths: A comparison of other-inflicted and self-inflicted shootings | Hemenway, 2010 | 2003-2006 | Unintentional death by firearm | To describe the circumstances of unintentional firearm deaths and compare/contrast with other-inflicted and self-inflicted shootings | Decedents of unintentional firearm-related incidents, excluding those involving Russian roulette and unknown circumstances | 363 deaths | Manual review | No | Insufficient details on relevant demographic and circumstantial data such as characteristics of victim and shooters |
| Child maltreatment fatalities in children under 5: Findings from the National Violence Death Reporting System | Klevens, 2010 | 2003-2006 | Child maltreatment (homicide, unintentional death) | To describe the distribution of child maltreatment fatalities in children under 5 by age, sex, race/ethnicity, type of maltreatment, and relationship to alleged perpetrator | Children under age 5 who died of child maltreatment | 1,374 deaths (600 attributed to maltreatment) | Manual review | No | NVDRS substantially under-ascertained child maltreatment; inconsistencies between different sources such as between C/ME and LE narratives and across states |
| Too many or too few unintentional firearm deaths in official U.S. mortality data? | Barber, 2011 | 2003-2006 | Unintentional death by firearm | To examine the accuracy of the NVDRS as a source of data on unintentional deaths by firearms in comparison to mortality data | Decedents of unintentional firearm-related incidents | 549 cases | Manual review | NVDRS data was compared with the NVSS, though there was not attempt to link the two databases due to a lack of identifiers and individual-level vital statistics data | Errors in coding/abstraction; the authors were unable to link the NVDRS to the NVSS data |
| Characteristics of elderly and other vulnerable adult victims of homicide by a caregiver: national violent death reporting system--17 U.S. states, 2003-2007 | Karch, 2011 | 2003-2007 | Homicide | To characterize victims, mechanism of injury, victim–suspect relationship, history of abuse, location of injury/death, and precipitating circumstances among homicides of vulnerable adults by caregivers | Homicide decedents aged 18+, C/ME or LE report indicated the suspect was the victim's caregiver | 68 victims | Manual review | No | Variability in completeness of information on precipitating circumstances; victim and perpetrator's illnesses were not verified by medical records |
| Sexual homicide and sexual violence-associated homicide: Findings from the national violent death reporting system | Smith, 2011 | 2003-2007 | Sexual homicide, sexual violence assoicated-homicide | To examine prevalence of sexual homicides and sexual violence–associated homicides and their precipitating circumstances | Decedants of homicides in which rape/sexual assault was precipitating circumstance or narratives contained sexual elements | 285 victims | Manual review | No | Motivations of the perpetrator were not known/documented in NVDRS |
| Race, urban context, and Russian roulette: Findings from the national violent death reporting system, 2003-2006 | Wasserman, 2011 | 2003-2006 | Russian roulette | To investigate urbanism as a moderator for the relationship between minority status and Russian roulette death. The authors hypothesized that the relationship was stronger in the more urban counties | Cases were male suicide decedents involving Russian roulette; controls were male suicide decedents who died from a gunshot wound to the head | 284 controls, 71 cases | Keyword search and manual review | No | None mentioned |
| Factors Influencing Subjects' Observed Level of Suicide by Cop Intent | Lord, 2012 | 2003-2008 | Legal intervention deaths | To examine the subject's degree of intent and factors that affect that degree of intent using the Lord and Sloop's (2010) revised model | Decedents of legal intervention deaths | 918 deaths | Manual review | No | None mentioned |
| Health problems and male firearm suicide | Hempstead, 2013 | 2003-2009 | Suicide | To examine male suicides, use of firearms, and physical health problems | Male suicide decedents in New Jersey | 3,413 deaths | Manual review | Used 2007 New Jersey hospital discharge data to obtain physical health and history of hospitalization; and 2002-2004 New Jersey BRFSS for general physical health, mental health, and gun ownership | None mentioned |
| Precipitating Circumstances of Suicide Among Youth Aged 10-17 Years by Sex: Data From the National Violent Death Reporting System, 16 States, 2005-2008 | Karch, 2013 | 2005-2008 | Suicide | To examine characteristics and precipitating circumstances of suicides among youth aged 10-17 years, including how these differed by sex of the decedent | Suicide decedents aged 10-17 years | 1,047 decedents | Manual review | No | Narrative data depended on the accuracy and completeness of original sources and circumstances reported to the NVDRS |
| Homicide-followed-by-suicide incidents involving child victims | Logan, 2013 | 2003-2009 | Homcide-suicide | To describe homicide-suicides of child victims and perpetrators, including life-stress related preceding circumstances and their actions in the time leading up to the incidents | Decedents under age 18 who died in homicide-suicide incidents, excluding perpetrators under age 18 | 129 incidents (129 perpetrators and 188 child victims) | Manual review | No | Details abstracted from narratives (e.g., mental health) were limited to the informants and thoroughness of the investigations/documentation; errors in coding/abstraction |
| Violent death among recently released prison inmates: stories behind the numbers | Scheyett, 2013 | 2006-2009 | Homicide, suicide, legal intervention | To describe the circumstances of formerly incarcerated people who died in a violent manner post-release | Decedents who were released from North Carolina (NC) prisons between 2006 and 2007 and died violently prior to 2009 | 164 decedents | Manual review | Linked to the North Carolina Offender Population Unified System dataset and death certificates. Data of prison releases from 2006-2007 was matched to death certificates and then 2006-2009 NC-VDRS using probabilistic matching | Insufficient details on relevant demographic and circumstantial data to provide true context around the incidents, e.g. neighborhood characteristics, relationship problems, nature of altercations resulting in violence, and mental health treatment |
| Police Responses in Officer-Involved Violent Deaths: Comparison of Suicide by Cop and Non-Suicide by Cop Incidents | Lord, 2014 | 2004-2008 | Legal intervention deaths | To compare the influence of suicide by cop and non-suicide by cop subjects' personal characteristics and actions on officers' use-of-force decisions | Decedents of legal intervention deaths | 508 deaths (of the 918 legal intervention deaths in the NVDRS that contained sufficient information for analyses) | Manual review | No | Limited information on relevant circumstantial data such as mental health history, drug addiction, and history of suicide attempts |
| Intimate partner homicide and corollary victims in 16 states: National violent death reporting system, 2003-2009 | Smith, 2014 | 2003 - 2009 | Intimate partner related violent deaths (homicide, homicide-suicide, legal intervention, undetermined intent) | To examine the frequency and characteristics of IPH and corollary victims | Decedents who died by homicide, homicide-suicide, legal intervention, or undetermined intent deaths that were related to IPPs, excluding jealousy or love triangle in unrequited interest, sex or drug trades, and mercy killing | 4470 victims, 3350 IPV-related incidents | Manual review | No | NVDRS did not have circumstance code to capture homicides related to self-defense; insufficient information to determine whether the victim-perpetrator relationship was current |
| Increase in suicides associated with home eviction and foreclosure during the US housing crisis: Findings from 16 national violent death reporting system states, 2005-2010 | Fowler, 2015 | 2005-2010 | Eviction and foreclosure-related suicide | To examine the frequency, characteristics, and precipitating circumstances of eviction and foreclosure-related suicides during the US housing crisis that started in 2006 | Suicide decedents who experienced a loss of housing or impending loss of housing (that was not related solely to behavioral or relationship reasons) within 12 months prior to death, was owner or a rent-paying tenant, and had eviction or foreclosure cited in C/ME and LE reports. Excluded those who expressed concern about eviction or foreclosure, but neither had occurred | 929 decedents | Keyword search | No | Circumstance information abstracted were limited to the accuracy and completeness of the C/ME and LE reports; significant missingness on circumstantial data |
| Children and unintentional firearm death | Hemenway, 2015 | 2005-2012 | Unintentional firearm deaths | To estimate the annual number of unintentional firearm deaths among children; describe the circumstances and shooters in such events; provide information on child shooters in all unintentional firearm deaths, including when children shoot adolescents and adults | Children aged 0 - 14 who were either victims or shooters in unintentional firearm deaths | 229 deaths | Manual review | Data from the BRFSS (2001, 2002 and 2004) was used and findings from a study (Kalesan et al. 2015) to examine household gun ownership | Many narratives lacked adequate and detailed information about the incident circumstances such as how the gun was accessed |
| Precipitating circumstances of suicide among active duty U.S. Army personnel versus U.S. civilians, 2005-2010 | Logan, 2015 | 2005-2010 | Suicide | To compare suicide events between active duty U.S. Army versus civilian decedents to inform military prevention efforts | Cases were active duty US Army decedents, excluding multi-victim suicide incidents; controls were civilian suicide decedents matched by sex, age, state and year of death | 141 active duty decedents, 563 civilian decedents | Manual review | Linked with the Department of Defense Suicide Event Reports | Details abstracted from narratives (e.g., mental health) were limited in the knowledge of the informants and the thoroughness of the investigations and documentation |
| Suicide risk in nursing homes and assisted living facilities: 2003–2011 | Mezuk, 2015 | 2003-2011 | Suicide | To study the epidemiology of suicide among adults in long-term care and assisted living facilities; assess how organizational characteristics of the facility affect risk; examine the role of anticipation of entry into a long-term care facility as a risk factor | Decedents aged 50+ who were residents of and died in Virginia from suicide or undetermined intent deaths, excluding incidents occurring in prisons or jails | 3,682 deaths (3,454 suicides and 229 undetermined deaths) | Manual review | Used the Nursing Home Compare (for long-term care facilities), VA state license records (for assisted living facilities), Nursing Home Compendium, and US Census | Narratives were not collected from clinical interviews, so mental health did not reflect psychiatric diagnosis; lack of information on demographic composition of facilities, clinical details about LTC utilization, or characteristics of the physical and social space |
| Acute and chronic risk preceding suicidal crises among middle-aged men without known mental health and/or substance abuse problems: An exploratory mixed method analysis | Schiff, 2015 | 2005-2010 | Suicide | To investigate suicide risk and the precipitating circumstances among middle-aged men, specifically of those without known mental health or substance abuse problems | Male suicide decedents aged 35-64 years who experienced a recent crisis, excluding those with mental health, alcohol, substance abuse problem or had history of suicide attempts | 600 decedents | Manual review | No | Variability in length and completeness of narratives by state and incident; narrative data was limited to the knowledge of the informants and may be biased because of the emotional context of the incidents |
| Improved Ascertainment of Pregnancy-Associated Suicides and Homicides in North Carolina | Austin, 2016 | 2005-2011 | Pregnancy-related suicide and homicide | To determine whether linking the NC-VDRS to traditional maternal mortality data allows for improved ascertainment and understanding of pregnancy-related violent deaths in the state | NC female residents aged 10-50 who died by suicide or homicide during pregnancy or postpartum periods between 2005-2011 | 84 incidents (29 suicide and 55 homicides) | Unclear | Linked with North Carolina's 'traditional' maternal mortality surveillance structure (i.e., death certificates, live birth and fetal death records, and hospital discharge records) | Narratives were collected from friends/family and limited to information known by C/ME and LE, e.g., autopsy could miss pregnancy in early stages |
| Identifying and Tracking Gas Suicides in the U.S. Using the National Violent Death Reporting System, 2005-2012 | Azrael, 2016 | 2005-2012 | Gas-specific suicide | To identify gas-specific suicides and track the incidence of these suicides over time by type of gas including carbon monoxide, helium, hydrogen sulfide, and others | Decedents of gas-specific suicides, excluding incidents with carbon monoxide inhalation as secondary cause | 3,242 deaths | Keyword search and manual review | No | Identifying gas-specific suicides using the NVDRS was laborious, inefficient and required active review of narratives; existing NVDRS variable “source of gas” was used infrequently |
| Homicides by police: Comparing counts from the national violent death reporting system, vital statistics, and supplementary homicide reports | Barber, 2016 | 2005-2012 | Legal intervention deaths | To evaluate the accuracy and completeness of the NVDRS as a method to identify homicides by LE | Decedents of homicides in which the suspect was a LE officer (including corrections officers), excluding legal executions | 1552 incidents | Manual review | Counts of homicide incidents in the NVDRS were compared with those in the CDC's vital statistics mortality data and the FBI's supplementary homicide reports | Many incident narratives were blank or uninformative; a substantial portion of homicides by police were missing due to coding errors and/or abstractors did not have access to information until a case was closed |
| Occupational Homicides of Law Enforcement Officers, 2003–2013: Data From the National Violent Death Reporting System | Blair, 2016 | 2003-2013 | Homicide | To describe the incidence and circumstances of homicides of LE officers, including the victim demographics and contexts in which their death occurred | LE officers killed in line of duty, excluding bail bondsmen, private security guards, and emergency responders | 128 victims, 121 incidents | Manual review | No | NVDRS only collected risk factor data and no protective factor data; data completeness and accuracy depended on the availability and/or quality of the original sources |
| Deaths Due to Use of Lethal Force by Law Enforcement: Findings From the National Violent Death Reporting System, 17 U.S. States, 2009–2012 | DeGue, 2016 | 2009-2012 | Legal intervention deaths | To describe the incident and victim characteristics of fatalities resulting from LE use of lethal force in order to inform risk assessment, training, and prevention policies | Decedents of legal intervention deaths or homicides in which the perpetrator was a LE officer | 812 deaths | Manual review | No | Narratives had limited details on relevant demographic and circumstantial data such as characteristics of the officers/perpetrators; biases in LE reports |
| Violent Deaths Among Georgia Workers: An Examination of Suicides and Homicides by Occupation, 2006–2009 | Lavender, 2016 | 2006-2009 | Homicide and suicide | To describe violent deaths among Georgia workers by occupation, including deaths occurring at work and outside of the workplace, and to identify precipitating circumstances for the occupations most at risk | Georgia workers aged 16+ who experienced a violent death, excluding homemaker, unemployed, disabled, student, child/ infant/toddler, self-employed, or unknown occupation | 4,616 deaths | Manual review | Used 2006–2009 Bureau of Labor Statistics Current Population Survey to obtain the number of employed persons in Georgia by occupation and the age and sex distribution of employed persons by occupation | Variations in abstractor coding of violent deaths; limited or insufficient information on certain circumstantial data such as work relationship/work related variables, suicide and homicide attempts or ideations |
| Mining for murder‐suicide: An approach to identifying cases of murder‐suicide in the national violent death reporting system restricted access database | McNally, 2016 | 2003-2010 | Homicide-suicide | To present a technical note on mining the NVDRS for murder-suicides that fosters replication and enables researchers to best identify true murder-suicide incidents | Decedents of murder-suicides in which both incidents occurred within 24 hours of one another | 107,707 incidents (1,519 of this sample was murder-suicides based on mining methods) | Manual review | No, though study linked qualitative and quantitative data within the NVDRS used a combination of year, state identification number, and incident identification number | None mentioned |
| Mixed Methods Analysis of Sex Differences in Life Stressors of Middle-Aged Suicides | Stone, 2016 | 2003 - 2011 | Suicide | To examine life stressors impacting middle-aged suicide; determine whether these stressors vary by sex; and explore their co-occurrence | Random sample of 630 suicide decedents aged 35-64 years with known circumstances of death | 630 decedents (315 women and 315 men) | Manual review | No | Limited circumstantial information; all were suicide decedents and no control group in NVDRS |
| Suicide among people with epilepsy: A population-based analysis of data from the U.S. National Violent Death Reporting System, 17 states, 2003-2011 | Tian, 2016 | 2003-2011 | Suicide | To describe the epidemiology of suicide among people with epilepsy; describe characteristics and the annual trend in this population; compare these characteristics and trend between people with and without epilepsy who died from suicide | Suicide decedents aged 10+. Cases are decedents with epilepsy; controls are those without epilepsy | 81,529 decedents without epilepsy, 972 decedents with epilepsy. Analysis of subset included 68,662 decedents without epilepsy and 833 with epilepsy | Keyword search and manual review | The 2007 National Survey of Children's Health was used to estimate the prevalence of epilepsy for children aged 10-17 years and the 2010 U.S. National Health Interview Survey to estimate the prevalence of epilepsy in adults in the general population | Narrative data depended on the knowledge of the informants about epilepsy; lack of medical history data |
| Youth firearm suicide: Precipitating/risk factors and gun access | Choi, 2017 | 2005-2014 | Suicide | To investigate how firearm use has changed relative to other means among youth suicide victims over time; how these deaths differ from those who used other means on various factors; what types of guns youth used, who owned the guns, and how did youth access the guns | Suicide decedents under age 21, excluding single or multiple homicides preceded the suicide and cases for which accidental vs. intentional self-harm were not clear from LE reports | 7,489 incidents (3,116 firearm) | Manual review | No | Narratives contained limited information about gun access, ownership, mental health disorders, toxicology, and precipitating circumstances; variations in data collection and quality of investigation across states |
| Methods and Findings from the National Violent Death Reporting System for Identifying Gang-Like Homicides, 2005-2008 | Frazier, 2017 | 2005-2008 | Gang-related homicide | To develop a "gang-like" homicide variable that can be applied to the NVDRS in order to more accurately capture gang-related homicides in the US and avoid regional/city variability | Decedents of “gang-related”homicides, excluding incidents where victim was intimate partner/friend, if the homicide resulted from a drug deal or drug use | 1,177 deaths (696 identified by the NVDRS variables, 481 from search by authors) | Manual review | No | Narrative data was limited to information available to the C/ME and LE, especially in incidents of ongoing investigations; insufficient circumstantial details in narratives to characterize gang-like homicides |
| The epidemiology of homicide perpetration by children | Hemenway, 2017 | 2005-2012 | Homicide | To describe the epidemiology of homicides that are perpetrated by children (age 0-14) and to use narrative and NVDRS information typology of events | Decedents of homicides in which suspects were children aged 0-14 years | 146 incidents, 154 suspects (151 victims) | Manual review | Used Vital Statistics data to determine the total number of children perpetrating homicide during the study period | Narrative data depended on the interpretations of the abstractors; limited data on important risk factors (e.g., prior gun carrying, hyperactivity, drug use, parenting, income inequality, neighborhood features) and demographics (e.g., income, education); NVDRS could not capture incidents with child perpetrator |
| Antecedents of Suicide among Youth Aged 11-15: A Multistate Mixed Methods Analysis | Holland, 2017 | 2003-2014 | Suicide | To identify common characteristics and antecedents of suicides among youth (age 11-15); and to contextualize suicidality among this age group | Random sample of 30% of suicide decedents aged 11-15 years | 482 decedents (30% random sample from total number for age group/time span) | Manual review | No | Information from narratives was limited the knowledge and willingness of the informants to share details about suicide incidents; variations in narrative data across states; lacked of information on sexual orientation |
| Military Versus Civilian Murder-Suicide | Patton, 2017 | 2003-2010 | Homicide-suicide | To compare motives, demographics, behavioral health factors, and incident characteristics of military versus civilian murder-suicides; and to evaluate which of these factors predict membership to military or civilian murder-suicide perpetrator groups | Decedents of murder-suicides occurred within 24 hours of one another, excluding dual homicides, female perpetrators, incidents where perpetrators were killed by LE, accidents, and incidents without C/ME and LE reports | 1,330 incidents | Manual review | No | Completeness and accuracy of narrative data were limited the knowledge of informants; limited demographic and circumstantial information such as military status, history of suicide attempts and mental health treatment |
| Factors associated with intimate partner homicide in Illinois, 2005–2010: Findings from the Illinois Violent Death Reporting System | Yousuf, 2017 | 2005 - 2010 | IPV related homicide | To investigate the circumstances surrounding the place of injury that led to the victim’s death in Illinois IPH cases | Decedents of homicides or homicide-suicides in which the perpetrator was identified as a spouse, current boyfriend/girlfriend, ex-partner, or other intimate partners | 275 incidents | Manual review | No | Variation in data collection surrounding the homicides; misclassification of the manner of death |
| Circumstances Preceding Homicide-Suicides Involving Child Victims: A Qualitative Analysis | Holland, 2018 | 2003-2011 | Homicide-suicide | To identify the contexts and precipitating circumstances of homicide-suicides involving children | Decedents of homicide-suicides involving child victims under age 18, excluding incidents where the perpetrators were under 18 or had unknown age | 175 incidents (175 perpetrators, 83 adult victims, 253 child victims) | Manual review | No | None mentioned |
| Characteristics of Victims and Suspects in Domestic Violence-Related Homicide - Rhode Island Violent Death Reporting System, 2004-2015 | Jiang, 2018 | 2004-2015 | Domestic violence related homicide | To examine the characteristics of victims and suspects (e.g., vulnerabilities of victims, suspect motivations) in domestic violent homicides | Homicide victims in Rhode Island between 2004 and 2015 | 354 cases (90 cases involved domestic violence) | Manual review | No | Limited information on suspects and toxicology tests; limited access to incidents that occurred in the state but the victims died in another state |
| Circumstances of suicide among individuals with a history of cancer | Massetti, 2018 | 2004-2013 | Suicide | To investigate and compare precipitating circumstances of suicide among decedents with and without a history of cancer | Cases were suicide decedents with a history of cancer; controls were suicide decedents with no cancer mentioned in narratives or cancer mentioned in irrelevant context | 90,581 incidents (4,182 with cancer history and 86,078 with no cancer history) | Keyword search | No | Information from narratives was limited the knowledge and willingness of the informants to disclose; limited information on cancer diagnosis (e.g., malignancy of tumors); variation in narrative data across states |
| Chronic pain among suicide decedents, 2003 to 2014: Findings from the national violent death reporting system | Petrosky., 2018 | 2003-2014 | Suicide | To investigate the burden of chronic pain among suicide decedents and to describe and characterize suicide decedents that suffered from chronic pain | Suicide decedents aged 10+ with chronic pain of at least 3 months, excluding emotional pain | 123,181 decedents (10,789 with evidence of chronic pain) | Keyword search | Used the US census data to calculate crude rates | Reliability and validity of medical diagnoses in narratives depended on knowledge of the informants; limited information on pain characteristics and factors associated with chronic pain (e.g., sleep disturbances, disability) |
| Incidence and Method of Suicide in Hospitals in the United States | Williams, 2018 | 2014 - 2015 | Suicide | To estimate the rate of inpatient suicides, method and location of suicides within hospitals in the US | Suicide decedents who died in hospitals, excluding incidents that occurred in nursing care centers, substance use rehabilitation centers, hospice facilities, involving nonpatients, and taking place after discharge | 46 incidents | Manual review | Used the Joint Commission's Sentinel Event Database and American Hospital Association Annual Hospital Survey to calculate the proportion of hospitals accredited by the Joint Commission | Lack or insufficient details in narratives about contextual factors, including ligature and ligature fixture point, or observation status of a patient at the time of the event; reliability of NVDRS codes for location of suicides; variation in data completeness and accuracy across states |
| Epidemiology of asphyxiation suicides in the United States, 2005-2014 | Yau, 2018 | 2005 - 2014 | Asphyxiation suicide | To describe characteristics of asphyxia suicide decedents; explore whether the decedents’ demographic characteristics were associated with suicide method neck compression, suffocation, chemicals or gasses) | Suicide decedents who died from one specific type of asphyxiation (e.g., smothering, hanging, strangulation, or chemicals), excluding deaths due to smoke inhalation and carbon monoxide | 25,270 deaths | Keyword search | Used US Census Bureau and National Center for Health Statistics for US bridged-race population estimates | None mentioned |
| Family Can Hurt You the Most: Examining Perpetrators in Multiple Casualty Events | Abolarin, 2019 | 2003-2015 | Multiple homicide | To explore possible associations and causations that precipitate multiple homicide events; to investigate relationships of the victims, suicidality of the perpetrator, and the role of IPV in multiple homicide cases | Decedents in homicide incidents involving 2 or more victims, excluding those with multiple perpetrators | 2,425 incidents (5,424 total victims) | Manual review | No | Limited relationship data; not all NVDRS states have adopted an intimate partner module |
| The Role of Intimate Partner Violence in Homicides of Children Aged 2-14 Years | Adhia, 2019 | 2005-2014 | Homicide | To examine how IPV-related child homicides differ from other child homicides; to investigate the differences between the coding of IPV-related cases by the NVDRS and narrative data in IPV-related cases | Children aged 2-14 years victims of homicides or legal intervention deaths | 1,386 victims | Manual review | No | Variability in the length of narratives by victim characteristics |
| Intimate Partner Homicide of Adolescents | Adhia, 2019 | 2003-2016 | Homicide | To determine the proportion of adolescent homicides perpetrated by an intimate partner; to describe the victims, perpetrators, and characteristics of these incidents | Homicide decedents aged 11-24 years | 8,048 victims (2,188 aged 11-18 years and 5,860 aged 19-24 years) | Manual review | No | Detail included in the narratives are limited to the accuracy and depth of C/ME and LE reports; lack of information about family histories and backgrounds for the victims |
| Suicide Among Persons With Dementia, Georgia, 2013 to 2016 | Annor, 2019 | 2013-2016 | Suicide | To investigate suicide among persons with dementia by describing characteristics and circumstances as well as risk factors of suicide in this population | Suicide decedents with diagnosed Alzheimer's and related dementias | 91 decedents for descriptive analyses and 141,592 for regression analyses | Keyword search | Linked data from the Georgia Alzheimer's Disease and Related Dementia registry to VDRS data for the State of Georgia and the Georgia Vital Records death data | Underestimation of suicides in the death certificates |
| Modifiable sleep-related risk factors in infant deaths in Cook County, Illinois | Briker, 2019 | 2015-2016 | Undetermined intent | To describe the characteristics of sleep-related infant deaths in Illinois, including the risk factors, locations, and demographics | Infants under 6 months of age who died in Cook County with manner of death classified as undetermined intent | 116 incidents | Manual review | No | Insufficient details on relevant circumstances such as family member cigarette use, housing stability, involvement with the Department of Child and Family Services, whether the family received food assistance or Medicaid support, family and medical histories |
| Intimate partner problems and suicide: are we missing the violence? | Brown, 2019 | 2005-2015 | IPV related suicide | To determine the proportion incidents where the circumstances were coded as IPP and IPV-related suicides and to determine if a significant number of suicides with IPV have only been identified as IPP within the NVDRS | Suicide decedents in Kentucky (including non-residents) | 5029 incidents | Manual review | No | Narrative data is dependent on the accuracy of C/ME and LE reports; recall bias was possible as circumstantial data was collected from family/friends; missingness on circumstantial data; NVDRS does not collect cyber bully |
| Suicide Decedents in Correctional Settings: Mental Health Treatment for Suicidal Ideation, Plans, and/or Attempts | Choi, 2019 | 2005-2014 | Suicide | To describe differences in demographics and risk factors for suicide decedents in correctional facilities, with focus on incarcerated individuals with history of suicidal ideation and/or attempts | Suicide decedents who were in custody (including police lockups and other detention facilities) at the time of death | 1,727 deaths | Manual review | No | Narratives were collected from family/friends; insufficient details about the circumstances of the suicides and whether the person was in a prison or jail |
| Physical Health Problems as a Late-Life Suicide Precipitant: Examination of Coroner/Medical Examiner and Law Enforcement Reports | Choi, 2019 | 2005-2014 | Suicide | To investigate how physical health problems (real or perceived) shaped suicidality in later life; to explore how decedents with physical health problems recorded as a precipitating circumstance differed from those for whom this was not recorded; and to identify frequently mentioned physical health problems and/or related concerns in C/ME and LE reports | Suicide decedents aged 65+, excluding those who were also suspects of multiple homicides and unknown age or sex | 111,534 incidents (16,924 aged 65 or older) | Keyword search and manual review | No | Contents of narratives depended on the interpretations and/or information deemed relevant by the abstractors; insufficient details to fully understand the circumstances of suicides; proxy-derived or suicide-note-based data on health problems could be questionable; no quantitative data on psychological risk factors; variations in availability and quality of the investigations across states |
| Validating the national violent death reporting system as a source of data on fatal shootings of civilians by law enforcement officers | Conner, 2019 | 2015 | Legal intervention | To assess the completeness of NVDRS counts of officer-involved killings of civilians compared against comprehensive open-source datasets. The authors also aimed to validate the NVDRS dataset's capture of these incidents that are firearm-related | Decedents of homicides killed by LE while in custody | 508 from the NVDRS (406 determined legal intervention homicides) and 675 from the open-source dataset. Deaths overlap since data are linked. | Manual review | Linked with five open-source datasets: FatalEncounters.org, Mapping Police Violence, The Counted, Gun Violence Archive, and The Washington Post | Miscoding legal intervention death as homicide or suicide; NVDRS cannot provide real-time information on legal intervention deaths; non-firearm-related legal intervention deaths are often labeled as accidental or from natural causes; incidents were not available to abstractors due to ongoing investigations |
| Correlates of Intimate Partner Homicide among Male Suicide Decedents with Known Intimate Partner Problems | Logan, 2019 | 2003-2015 | IPP related homicide | To investigate circumstances of IPH perpetration among male suicide decedents who had known IPPs | Male suicide decedents aged 18+ who killed current or former intimate parner(s) and had IPPs as precipitating circumstance | 30,259 decedents | Manual review | No | Narratives are limited to the completeness and accuracy of the C/ME and LE reports; variations in coding based on the abstractor's experience; limited data on medical/mental health |
| Suicides Among Lesbian and Gay Male Individuals: Findings From the National Violent Death Reporting System | Lyons, 2019 | 2003-2014 | Suicide | To describe suicide epidemiology among lesbian and gay male individuals | Suicide decedents aged 15+; LGBT subgroup was decedents self-identified as LGBT, perceived to be LGBT by family or their social network, or identified as transgender upon autopsy | 122,383 decedents classified as non-LGBT and 621 classified as LGBT | Keyword search | No | NVDRS data was limited by the availability, completeness, and accuracy of the original sources; narratives lacked details on intimate relationships; possible misclassification of decedents' sexual orientation |
| Suicide Among Older Adults Living in or Transitioning to Residential Long-term Care, 2003 to 2015 | Mezuk, 2019 | 2003-2015 | Suicide | To estimate the number of suicides associated with residential LTC and to characterize deaths associated with LTC compared with other suicide decedents. The study also aims to improve the data quality of suicide surveillance systems | Decedents aged 55+ who died by suicides, accidental, or undetermined intent deaths, excluding those with missing C/ME and LE narratives | 47,759 decedents (1037 associated with LTC) | Natural language processing | No | Completeness and accuracy depended on the availability and quality of original sources, experience of abstractors, etc; NVDRS was unable to capture suicide attempts |
| Ten-Year Trend and Correlates of Reported Posttraumatic Stress Disorder among Young Male Veteran Suicide Decedents-Results from the National Violent Death Reporting System, 16 U.S. States, 2005-2014 | O'Donnell, 2019 | 2005 - 2014 | Suicide | To examined trends and correlates of reported post-traumatic stress disorder among young male Veteran suicide decedents | Male Veterans aged 18–34 years who died by suicides | 1,362 decedents | Keyword search and manual review | Used Veteran Population Projection Model Data produced by the Department of Veterans Affairs to estimate the male Veteran population | Completeness and accuracy depended on the availability and quality of original sources (C/ME and LE, toxicology, vital statistics); no medical records in NVDRS |
| An Investigation of the LGBTQ+ Youth Suicide Disparity Using National Violent Death Reporting System Narrative Data | Ream, 2019 | 2013-2016 | Suicide | To identify new or underresearched risk factors for suicide risk among LGBTQ+ youth; to explain why the LGBTQ+/non-LGBTQ+ suicide disparity narrows into adulthood | Suicide decedents aged 12-29 years; LGBTQ+ decedents were matched to non-LGBTQ+ of the same biological sex | 788 decedents (394 LGBTQ+ cases matched with 394 non-LGBTQ+ cases) | Manual review | Used data from the CDC to obtain urbanicity | Blank or uninformative narratives; coding and abstraction is subject to human interpretation and errors; reliability of sexual orientation data in NVDRS |
| What's Unique About Lesbian, Gay, Bisexual, and Transgender (LGBT) Youth and Young Adult Suicides? Findings From the National Violent Death Reporting System | Ream, 2019 | 2013-2015 | Suicide | To describe suicides of LGBT youth and young adults and compare circumstances by sexual orientation, primarily between LGBT and non-LGBT cases and among LGBT subgroups | Suicide decedents aged 12-29 years, excluding those who had missing or unknown values for sexual orientation and were not coded as transgender | 10,311 decedents (2,209 of those coded as LGBT) | Keyword search | Used data from the CDC to obtain urbanicity | Completeness and accuracy depended on the availability and quality of original sources (C/ME and LE, toxicology, suicide notes); lacked data on the decedents' sexual orientation |
| Honor-Related Suicide in the United States: A Study of National Violent Death Reporting System Data | Roberts, 2019 | 2003-2011 | Honor-related suicide | To apply a systematic method to describe honor-related suicides as they relate to negative life events, levels of personal distress, treatment-seeking behaviors for mental health issues, and how one is communicating suicidal intent/motivations, and how honor-related suicides compare to non honor-related suicides on these issues | Suicide decedents aged 18+; honor-related cases were suicides explicitly related to a victim's concerns about personal honor and experience of shame | 54,333 cases (163 of these determined as honor-related by authors' narrative review) | Keyword search and manual review | No | Variation in narrative details; insufficient information about the circumstances of negative life events (only the presence or absence of certain factors was recorded) |
| Characteristics and Precipitating Circumstances of Suicide Among Incarcerated Youth | Ruch, 2019 | 2003-2012 | Suicide | To describe the circumstances of suicides of incarcerated youth and compare them with those of non-incarcerated youth and youth in the general population | Suicide decedents aged 10-24 years, excluding those with missing circumstances precipitating suicide. Incarcerated youth were those in custody at the time of suicide | 10,126 decedents (9,913 not in custody and 213 in custody) | Manual review | No | Narratives for youth died of suicide in custody were not consistently available; insufficient data on suicide method, location, duration of confinement, criminal history, precipitating circumstances, toxicology, psychopathology and suicide risk factors |
| Characteristics and contextual stressors in farmer and agricultural worker suicides in Georgia from 2008–2015 | Scheyett, 2019 | 2008-2015 | Suicide | To describe characteristics and risk factors of suicides of farmers and agricultural workers in the US context | Farmers or agricultural workers who died by suicides between 2008-2015 in Georgia | 106 decedents | Manual review | No | Completeness and accuracy depended on the availability and quality of original sources; C/ME and LE reports varied by individual investigator/investigating unit; inconsistencies in classifying farmers and agricultural workers |
| Circumstances preceding suicide in U.S. soldiers: A qualitative analysis of narrative data | Skopp, 2019 | 2005-2010 | Suicide | To describe the circumstances surrounding military suicides | Active duty US army decedents | 135 decedents | Manual review | Linked with the Department of Defense Suicide Event Reports to identify active duty US army decedents and aggregate narrative data | Completeness and accuracy of precipitating circumstances depended on the availability and quality of original sources; suicides were possibly misclassified as accidental or of undetermined cause |
| Unintentional firearm deaths in the United States 2005–2015 | Solnick, 2019 | 2005 - 2015 | Unintentional firearm-related deaths | To describe the circumstances of unintentional firearm deaths in the US | Decedents of unintentional firearm deaths, excluding suicides due to a pre-existing medical condition | 1,260 incidents | Manual review | The number of gun fatalities from NVDRS was compared with other sources (e.g., Vital Statistics) | There was a lack or insufficient details in narratives to characterize circumstances of incidents, particularly how guns were accessed, whether hunters pursued big or small game, how often circumstances were missed or misreported |
| Timing of suicide in people with epilepsy: A population-based study from 18 states of the United States, 2003-2014 | Tian, 2019 | 2003-2014 | Suicide | To distinguish suicide timing patterns in those with and without epilepsy, discuss reasons for these patterns, and provide prevention measures | Suicide decedents aged 10+ | 1,310 decedents with epilepsy and 102,582 decedents without epilepsy | Keyword search and manual review | Used US Census Bureau and CDC's WONDER to estimate counts of the population aged 10+, which were then used to calculate suicide rates and rate ratios in persons with and without epilepsy | None mentioned |
| Murder-Suicides Perpetrated by Adolescents: Findings from the National Violent Death Reporting System | Adhia, 2020 | 2003 - 2016 | Homicide-suicide | To characterize murder-suicides perpetrated by adolescents while using Joiner’s (2014) perversion of virtue framework | Decedents of single or multiple homicides followed by suicides in which the perpetrators were under 21 years old | 47 incidents (56 victims) | Manual review | No | Insufficient details in narratives to classify incidents based on motivation and intent; variation in narrative data between incidents |
| A multi-state examination of the victims of fatal adolescent intimate partner violence, 2011-2015 | Bush, 2020 | 2011-2015 | Initimate partner violence related violent death (homicide, homicide-suicide, legal intervention death, undetermined intent) | To describe fatal IPV-related incidents where the decedent was an adolescent. The authors aim to characterize the precipitating circumstances | Dededents aged 15-19 years who died by homicides, homicide-suicides, legal intervention deaths, or undetermined intent deaths with following circumstances: IPP, jealousy or love triangle, other argument, conflict or abuse, or the victim-suspect relationship being an intimate partner | 93 incidents (116 decedents) | Manual review | No | Completeness and accuracy depended on the availability and quality of original sources; variations in data reporting across states, researchers, and practitioners due to different IPV terminology; missingness in pregnancy status, toxicology, relationships variables |
| Prevalence of Bullying among Youth Classified as LGBTQ Who Died by Suicide as Reported in the National Violent Death Reporting System, 2003-2017 | Clark, 2020 | 2003-2017 | Suicide | To investigate whether bullying is a common antecedent circumstance among LGBTQ youth who die by suicide | Suicide decedents aged 10-19 years; LGBTQ+ cases were those self-identified as LGBTQ, perceived to be LGBTQ by family/friends/peers, or accessed treatment for gender dysphoria | 9,884 decedents (334 classified as LGBTQ status and 490 as bullying status) | Keyword search | No | LGBTQ information was not systematically reported in death records |
| Resident-to-Resident Aggression in Long-Term Care: Analysis of Structured and Unstructured Data From the National Violent Death Reporting System, 2003-2016 | DeBois, 2020 | 2003-2016 | Homicide | To assess the ability of the NVDRS to capture resident-to-resident aggression (RRA) fatalities. The authors examined the extent to which their findings supported previous research, and provided recommendations for the continued analysis of RRA trends and interventions | Decedents aged 65+ of homicides that occurred in a long term care facility and both victim and exhibitor were residents of the facility | 101 incidents | Manual review | Used supplementary media reports, when available, to identify additional incidents of fatal RRA aggression | Limited demographic and diagnostic data (e.g., type of facility) for many resident-to-resident cases; NVDRS could not capture unrecorded aggression/fatalities, only cases reported by LE |
| Suicides Among Incarcerated Persons in 18 US States: Findings From the National Violent Death Reporting System, 2003–2014 | Dixon, 2020 | 2003-2014 | Suicide | To describe circumstances and characteristics of suicides among incarcerated persons, and compare them with suicides of nonincarcerated persons | Suicide decedents aged 10+ who were fatally injured while detained at a LE or correctional facility | 123,408 decedents (1,896 incarcerated and 121,512 nonincarcerated) | Manual review | No | Completeness and accuracy depended on the availability, quality of original sources, interpretations and/or information deemed relevant by the abstractors; toxicology reports were not available |
| Fatalities related to intimate partner violence: Towards a comprehensive perspective | Kafka, 2020 | 2010-2017 | IPV related violent death (homicide, suicide, homicide-suicide, legal intervention deaths, deaths of corollary victims) | To examine the burden of IPV to violent deaths and characterize commonalities across decedents and examine characteristics associated with homicide and suicide subtypes and legal intervention deaths | North Carolina victims of violent deaths who were in an abusive relationship (either as a victim or perpetrator), excluding deaths with unknown circumstances, undetermined intent or unintentional deaths | 1,470 deaths | Manual review | No | Narratives lacked details to characterize IPV; incident information was compiled from secondary sources (e.g., C/ME and LE); mental health, trauma history, and IPV variables were often missing |
| Age-and sex-specific risk factors for youth suicide: A mixed methods review | Kohlbeck, 2020 | 2012-2016 | Suicide | To identify risk factors by two age groups (i.e., pre-high school and high school) and by sex | Wiscosin residents aged 10-17 years who died by suicide between 2012-2016, excluding murder-suicides | 146 deaths | Manual review | No | Narratives were limited to the information provided from family/friends; race was determined by C/ME reports; no control group, all suicide decedents in NVDRS |
| Firearms and Protective Orders in Intimate Partner Homicides | Lyons, 2020 | 2003-2018 | IPH | To describe the use of protective orders among intimate partner victims and corollary victims of IPH and whether a firearm was used in the incident | Decedents who died by intimate-partner homicides, including both opposite and same-sex partners | 8,375 incidents (9130 victims) | Keyword search and manual review | No | Information on protective orders related to IPV were limited to the information provided by the source documents and interpretations of the abstractors; narrative contents did not include firearm removal; missing data on perpetrator and victim characteristics |
| Characteristics of interpersonal conflicts preceding youth suicide: analysis of data from the 2017 National Violent Death Reporting System | Orlins, 2020 | 2017 | Suicide | To examine characteristics of interpersonal conflict preceding suicides among youth | Decedents under age 17 who died by suicides within 24 hours or up to two weeks following an interpersonal conflict | 197 cases | Manual review | No | Narrative accounts of the events and related circumstances might have been biased |
| A test for differential ascertainment in case-control studies with application to child maltreatment | Sordello, 2020 | 2010 - 2015 | Child maltreatment (all violent deaths) | To understand whether deaths caused by child maltreatment are differentially ascertained by race | Children under age 10 whose deaths were caused by child maltreatment | 968 deaths | Manual review | No | Pre-coded NVDRS variables (ICD codes, LE_DeathAbuse and CME_DeathAbuse) were not enough to capture child maltreatment incidents |
| Violence As a Direct Cause of and Indirect Contributor to Maternal Death | Wallace, 2020 | 2011-2015 | Pregnancy-associated homicide | To examine how violent contexts are associated with increased risk of pregnancy-related mortality and pregnancy-associated homicide and how this risk varies by trends in violent crime in the US | Women who died by homicides and were pregnant at time of death or pregnant within the past year prior to death | 1,791 maternal decedents (174 violent deaths from NVDRS [113 pregnant, 53 postpartum] and 1,617 nonviolent) | Keyword search and manual review | The National Center for Health Statistics data was used to obtain pregnancy-related deaths (matched with NVDRS by states and period) and state-level live births. The Federal Bureau of Investigation's Uniform Crime Reporting Program database was also used to obtain annual counts of violent deaths. | Narrative data depended on the accuracy of C/ME and LE reports; underascertainment of cases where women were in early pregnancy, lost the infant or custody of the infant |
| A Typology of Civilians Shot and Killed by US Police: a Latent Class Analysis of Firearm Legal Intervention Homicide in the 2014–2015 National Violent Death Reporting System | Wertz, 2020 | 2014 - 2015 | Legal intervention | To construct an exhaustive and mutually exclusive typology of firearm LIH using identified incidents using a validated case identification process | Homicide decedents killed by LE officers while in custody | 603 incidents | Manual review | Used the 2015 American Community Survey to obtain characteristics of the US general population and CDC’s WISQARS online database for US general population's age distribution | Classification and characterization of legal intervention deaths depended on C/ME determination; variations in narrative details between incidents; limited information about the officer(s) who inflicted the fatal injury |
| Integrating topic modeling and word embedding to characterize violent deaths | Arseniev-Koehler, 2021 | 2003-2017 | All violent deaths (suicide, homicide, undetermined intent, unintentional death, legal intervention) | To identify latent topics in the narrative data and examine gender bias among the latent topics, including long guns (e.g., rifles and shotguns) and sedative and pain medications | All NVDRS decedents | 307,249 deaths; Analytic sample limited to 272,974 deaths with at least 50 words in the narrative. | Topic modeling | No | None mentioned |
| Aggression, Escalation, and Other Latent Themes in Legal Intervention Deaths of Non-Hispanic Black and White Men: Results From the 2003‒2017 National Violent Death Reporting System | Arseniev-Koehler, 2021 | 2003-2017 | Legal intervention | To find indicators of threat and dangerousness (e.g., physical aggression) and examine whether these themes played a role in the increased risk of death from legal intervention encounters among Black men | Male decedents aged 12+ who died by legal intervention deaths, excluding those in which the victim was a LE officer | 4,981 deaths | Topic modeling | Linked with the Agency for Healthcare Research and Quality's database on Social Determinants of Health for county-level violent crime rate and proportion of non-White county residents | Accuracy of narratives depended on type of death, accuracy/availability of original documents, and errors in completing narrative/death record; inconsistencies in NVDRS data |
| Suicides at shooting ranges | Barber, 2021 | 2004-2015 | Suicide | To examine the incidence of suicides at shooting ranges and describe the victim characteristics | Decedents of firearm suicides that occurred at a shooting range | 118 cases | Manual review | No | Level of detail of the incident narratives depended on completeness and detail of C/ME and LE reports |
| Health Care Worker Violent Deaths in the Workplace: A Summary of Cases From the National Violent Death Reporting System | Braun, 2021 | 2003-2016 | All violent deaths | To describe violent deaths of health care workers in the workplace, including the type of violent death, prevalence, and location | All NVDRS decedents | 61 deaths | Manual review | No | Missing incidents were due to underreporting of the NVDRS; many deaths were possibly from among patients and/or visitors though not easily identifiable from NVDRS |
| Exploring nurse suicide by firearms: A mixed‐method longitudinal (2003–2017) analysis of death investigations | Davidson, 2021 | 2003 - 2017 | Suicide by firearm | To understand the longitudinal incidence of nurse suicide by firearms compared to non-nurses, whether this incidence is changing, and whether topic modeling strategies could be utilized to focus content analysis of narratives | Suicide decedents over age 21 | 739 nurse deaths and 94,838 other deaths | Topic modeling | No | Variables on gun ownership, stored loaded, or stored locked were not consistently collected; insufficient information to characterize deaths among military decedents |
| Job-Related Problems Prior to Nurse Suicide, 2003-2017: A Mixed Methods Analysis Using Natural Language Processing and Thematic Analysis | Davidson, 2021 | 2003 - 2017 | Suicide | To examine job-related problems experienced before nurse death by suicide | Nurses aged 21+ who died by suicides related to problems in the workplace prior to death | 203 deaths | Manual review | No | Missing narratives; biases as data were collected from tertiary sources (family, employers, C/ME, LE) |
| Examining differences between mass, multiple, and single-victim homicides to inform prevention: findings from the National Violent Death Reporting System | Fowler, 2021 | 2003 - 2017 | All homicide (mass, multiple, single) | To examine characteristics of mass, multiple, and single homicides, including suspect demographics, mental health and suicidal behavior of perpetrators, location, weapons used, number of persons nonfatally shot, precipitating circumstances, and incident resolution | All homicide decedents | 728 victims of mass homicide killed in 141 incidents, 7,112 victims of multiple homicide killed in 3,439 incidents, and 74,623 victims of single homicide | Manual review | Linked with other databases for number of non-fatally shot persons within an incident (Gun Violence Archive, Mother Jones’ Mass Shootings Database, Everytown for Gun Safety mass shootings report, NYPD active shooter report and its updates/ appendices, and the FBI active shooter report) | Limited data for areas where data-sharing between different partners (vital records, C/ME, LE) were not fully developed; limited information about suspects and suspect motivations; medical/mental health did not come from medical records; completeness of narratives varies |
| Disparities in potential years of life lost due to intimate partner violence: Data from 16 states for 2006–2015 | Graham, 2021 | 2006-2015 | Intimate partner-related violent deaths (homicide, suicide, legal intervention) | To estimate the societal costs of IPH, IPH-suicides, and fatalities resulting from LE intervention in IPV in terms of potential years of life lost for partners and corollary victims. The study describes these fatalities by demographics | Decedents killed by an intimate partner, during an IPV, resulting from LE intervention in an IPV, or suicides that occurred after killing an intimate partner, excluding unintentional and undetermined intent deaths | 7,916 death (6,282 partner, 1,634 corollary) | Manual review | Used data on US average life expectancy from the National Center for Health Statistics | Misclassification of IPV incidents by NVDRS, particularly suicides that were not part of a homicide-suicide; errors in the NVDRS as a whole and narratives; limited data on gender non-conforming individuals’ experiences with IPV-related fatality |
| Intimate Partner Violence–Related Fatalities Among US Youth Aged 0–24 Years, 2014–2018 | Graham, 2022 | 2014-2018 | IPV related violent deaths (homicide, homicide−suicide, legal intervention, suicide) | To understand the proportion of violent deaths among US young people where IPV is a precipitating factor, whether these deaths differ by age, sex, race/ethnicity, and the proportion that are perpetrated using a firearm versus another weapon | Decedents aged 0-24 years who died by IPV-related deaths | 1,927 incidents | Manual review | Used the US Census to calculate an IPV-related fatality rate (per 100,000 person years) | Limited data on corollary victims, sexual orientation; challenges to determine if the decedent was a perpetrator or victim; inconsistencies in coding and details of circumstantial variables related to IPV; missingness |
| Understanding Escalation Through Intimate Partner Homicide Narratives | Kafonek, 2021 | 2014-2017 | IPH | To explore the escalating circumstances leading up to a IPH where prior abuse by the suspect was affirmed among women | Women decedents of IPHs | 1,148 cases (those with some aspect of escalation) | Manual review | No | Variation in narrative details; lacked data on risk/lethality assessments and whether the victim received risk assessment; missing C/ME and LE narratives |
| A comparison of farming- and non-farming-related suicides from the United States' National Violent Deaths Reporting System, 2003-2016 | Kennedy, 2021 | 2003 - 2016 | Suicide | To examine differences in sociodemographic and precipitating events between farming and non-farming suicide decedents | Suicide decedants aged 16+; farmers are individuals who are farm owner/manager/self-employed, farmworker/laborer, or no longer farming due to retirement/unemployment/disability | 140,523 deaths (2,801 farming-related and 137,722 non-farming) | Manual review | No | Occupational data could not distinguish between farm owners, managers, workers or identify members of farming families; farming-related risk factors were not collected; mental health was not based on structured interviews |
| A Data Science Approach to Estimating the Frequency of Driving Cessation Associated Suicide in the US: Evidence From the National Violent Death Reporting System | Ko, 2021 | 2003 - 2017 | Driving cessation associated suicide | To estimate the frequency of driving cessation associated suicide among aging adults, describe the salient circumstances, and discuss the utility of a supervised random forest algorithm in identifying these deaths | Decedents aged 55+ who died by suicides and undetermined causes | 65,942 incidents (59,080 suicides and 6,862 undetermined deaths) | Natural language processing | Used the 2013 county-level Rural Urban Continuum Code to proxy the relative reliance on automobiles for transportation | Heterogeneity in written descriptions of driving cessation; narratives were limited to information deemed relevant by the abstractors; NVDRS consolidated “No,” “Not Available,” and “Unknown” for certain variables |
| State Variation in Long-Term Care Availability, Regulation, and Cost and Suicide Mortality Among Older Adults in the United States: 2010-2015 | Lohman, 2021 | 2010 - 2015 | Suicide | To understand how state characteristics regarding availability, cost, and regulation of residential LTC vary across states and whether such variability associated with suicide mortality among older adults | Suicide decedents aged 55+ | 25,040 deaths (382 associated with long-term care) | Natural language processing | Used the National Study of Long-Term Care Providers to obtain availability of long-term care providers, data from insurance company Genworth for cost of long-term care, Trinkoff et al. for regulation data and the American Community Survey for state characteristics | Coded variables did not accurately indicate the decedents' locations and whether a decedent was transitioning into or out of long-term care at time of death |
| Risk Factors for Child Death During an Intimate Partner Homicide: A Case-Control Study | Lyons, 2021 | 2003-2017 | IPH | To understand risk factors for children corollary victims dying in IPH | Cases were decedents of IPH incidents with at least one child corollary victim younger than 18; controls were decedents of IPH incidents where corollary child victim was present but not killed | 968 victims and 577 incidents (227 case incidents and 350 controls) | Keyword search and manual review | No | Missingness in relevant risk factors, e.g., characteristics of the children in IPHs who were present, but not killed, in narratives |
| A cross-sectional study of opioid involvement in non-poisoning suicide–risks and prevention opportunities | Mason, 2021 | 2015-2017 | Non-posoining suicide | To examine the role that opioid use in suicide incidents where the cause of death is anything other than poisoning | All suicide decedents who had available toxicology report and cause of death was not poisoning or overdose. | 1,007 decedents (842 opioid-negative and 165 opioid-positive) | Manual review | No | Narrative information were from third parties and did not completely and accurately reflect the details of the incident; missing toxicology screening for opioids |
| Suicide Completion Among Incarcerated Women | Mennicke, 2021 | 2003-2015 | Suicide | To describe the circumstances of completed suicides among incarcerated women; compare the circumstances to those of incarcerated men; use qualitative thematic analysis methods with narrative data to identify further circumstances | Suicide decedents who were incarcerated in prison or jail | 2,104 decedents (176 female decedents and 1,928 male decedents) | Manual review | No | Inconsistencies and misclassification of prison and jail settings; variations in timing and data collection procedures across states; lacked information on circumstances relating to incarceration |
| Not discussed: Inequalities in narrative text data for suicide deaths in the National Violent Death Reporting System | Mezuk, 2021 | 2003-2017 | Suicide and undetermined death | To understand how narrative length and missingness varied as a function of decedent characteristics | Decedents of suicides or undetermined deaths, excluding incidents of multiple deaths and accidental firearm deaths | 233,108 deaths | Other; the authors reviewed the missingness and length of narratives | No | Variations in narrative length depended on the abstractors's experience, data-sharing between stakeholders, and decedent characteristics; source documents provided to the NVDRS were not designed for researchers |
| Child maltreatment fatalities among children and adolescents 5–17 years old | Michaels, 2021 | 2006 - 2015 | Child maltreatment (homicide, undetermined death) | To describe child maltreatment fatalities among children 5−17 years old | Children aged 5-17 years who died by homicides and undetermined deaths; child maltreatment were cases resulting from an intentional act or gross negligence of a parent or caregiver, excluding IPV | 285 incidents | Manual review | No | Inconsistencies in C/ME and LE narratives; NVDRS coding errors; missing variables on victim’s history of abuse; variation in length of narratives |
| Reported History of Traumatic Brain Injury Among Suicide Decedents: National Violent Death Reporting System, 2003-2017 | Miller, 2021 | 2003 - 2017 | Suicide | To examine differences in the characteristics of suicide decedents with and without a documented TBI history in the general population and those who ever served in the military | Suicide decedents aged ≥10 years with a documented history of traumatic brain injury prior to time of suicide | 993 incidents | Keyword search and manual review | No | Availability and completeness of narratives vary by state and jurisdiction; narratives relied on information from family/friends; lacked data on severity and risk of previous TBI, suicide attempts, and circumstances related to TBI |
| Integrating National Violent Death Reporting System Data into Maternal Mortality Review Committees | Miller, 2021 | 2014 - 2017 | Pregnancy-associated homicide and suicide | The study aims to examine characteristics of pregnancy-associated maternal deaths | Women who died by homicides or suicides and were pregnant at the time of death or within the previous 365 days | 520 deaths | Manual review | No | Narratives lacked details to determine pregnancy status; underidentification of suicide by C/ME |
| Characteristics and Factors Associated With Intimate Partner Violence–Related Homicide Post-Release From Jail or Prison | Rizo, 2021 | 2003 - 2015 | Intimate partner violence related homicide | To examine circumstances surrounding IPV-related homicides of individuals recently released from jail or prison and how and characteristics differed by relation to IPV | Decedents who died by homicides within 1 month of release from jail, prison, or a detention facility | 126 homicides (109 non-IPV-related and 17 IPV-related) | Manual review | No | Insufficient details to determine whether a particular case was related to IPV; missingness in homicide perpetrator characteristics; NVDRS only captured homicides that occurred within a month post-release |
| Characteristics and Precipitating Circumstances of Suicide Among Children Aged 5 to 11 Years in the United States, 2013-2017 | Ruch, 2021 | 2013-2017 | Suicide | To investigate circumstances and precipitating factors of childhood suicides | Children aged 5-11 years who died by suicides | 134 decedents | Manual review | No | Completeness and accuracy of narrative data varies by state and incident and was limited by the completeness and accuracy of the supporting documents; misclassifications of suicide incidents involving children |
| Comparisons between suicide in persons with serious mental illness, other mental disorders, or no known mental illness: Results from 37 U.S. states, 2003-2017 | Schmutte, 2021 | 2003-2017 | Suicide | To compare the characteristics of suicide decedents with serious mental illness with those with other mental disorders or no known mental illnesses | Suicide decedents aged 18+, excluding unintentional or undetermined deaths; decedents with serious mental illness were those diagnosed with schizophrenia or bipolar disorder | 174,001 decedents (8.7% with SMI; 33% with other mental disorders; 58.2% with no known mental illness) | Keyword search | No | Mental health was not based on standardized interviews; completeness and accuracy depended on the availability, quality of original sources; lacked clinical data (e.g., period of recovery, active psychosis) and toxicology |
| Homicide-suicide across the lifespan: a mixed methods examination of factors contributing to older adult perpetration | Schwab-Reese, 2021 | 2013-2016 | Homicide-suicide | To study individual and contextual factors of homicide-suicide for young adult (18-44 years), middle-aged adults (45-64 years), and older adults (65+ years) using a mixed methods approach | Decedents of single or multiple homicides followed by suicides in which the perpetrators were 18 years or older, excluding incidents with multiple perpetrators | 1,140 incidents (1,140 perpetrators, 1,368 total victims); For qualitative analysis, 179 older adult perpetrators that resulted in the deaths of 189 victims | Manual review | No | Narrative data depended on information available in the C/ME and LE reports and from family/friends; errors in data abstraction |
| Characteristics and patterns of older adult homicides in the United States | Shawon, 2021 | 2003-2017 | Homicide | To examine differences by injury mechanisms and compare circumstances of homicides in older adults as well as those that involved and did not involve firearms | Homicide decedents aged 60+ | 5961 incidents, 6188 total victims | Manual review | Victim characteristics were compared between NVDRS and WISQARS to generate age-adjusted rates | Completeness and accuracy of narrative data depended on information contained in the C/ME and LE reports; missingness in circumstantial variables such as race/ethnicity, mental illness, marital status, and victim-perpetrator relationship |
| Criminal histories of intimate partner homicide offenders | Zeoli, 2021 | 2014 - 2015 | IPH | To examine the criminal histories of male and female IPH offenders in Michigan | Decedents of IPHs in Michigan, excluding incidents where a third party committed the homicide, offender under age 18, self-defense or in which victim previously abused the offender | 117 cases, 103 offenders | Manual review | Linked to criminal justice records, including arrests, charges, and court disposition records, supplied by the Michigan State Police | Lacked data on domestic violence restraining orders for the IPH offenders |
| Homicide or Suicide: How Nudity Factors into This Determination | Craun, 2022 | 2014-2017 | Homicide and suicide | To explore “nudity: as a distinguishing factor between homicides and suicides; investigate interaction effect of victim gender and nudity on the likelihood of a death being a homicide or a suicide | Decedents who died by suicides or homicides and were found nude or partially nude | 119,145 deaths | Keyword search and manual review | No | Completeness and accuracy of narrative data depended on information contained in C/ME and LE reports; NVDRS collapses no/unknown/not available into one category; coding methods did not capture autoerotic fatalities |
| Differences Between Sexual and Nonsexual Homicides of Women in the United States: Findings From the National Violent Death Reporting System | De Veauuse Brown, 2022 | 2015 - 2018 | Homicide | To explore differences in offender, victim, and incident characteristics between sexual homicide and non-sexual homicides | Women aged 20-64 who died by homicides | 324 sexual homicide and 6137 non-sexual homicide; Among those with known precipitating circumstance, 297 sexual homicide and 5282 non-sexual homicide | Keyword search and manual review | No | Circumstantial information (e.g., alcohol use, pertinent victim, suspect, incident characteristics) may have been missing/incomplete due to ongoing investigations |
| Physician suicide in the united states: Updated estimates from the national violent death reporting system | Gold, 2022 | 2010-2015 | Suicide | To identify and describe physician suicides; compare physician suicides with non-physician suicides; and estimate national rates of physician suicides | Suicide decedents aged 27+, with sufficient information to determine occupation | 63,422 cases (357 were physicians) | Manual review | Data from the American Medical Association used to identify baseline population and calculate average rate of change from 2010-2013 by specific factors and estimate rate for 2014-2015. CDC WONDER used to obtain yearly population estimates. | Misclassification of deaths (e.g., gunshot and hanging deaths were more likely to be coded as suicides than drug intoxication deaths, which were often listed as 'undetermined'); coroners may have been less likely to declare a physician death to be a suicide due to stigma |
| An examination of fatal child poisonings in the United States using the National Violent Death Reporting System (NVDRS), 2012–2017 | Hunter, 2022 | 2012-2017 | Poisoning (homicide, unintentional or undetermined intent) | To examine the characteristics, toxicology, and modalities of fatal child poisonings. Authors hypothesized that the majority of fatal poisonings would be related to neglect, and involve prescription or illicit opioids | Children aged 0-9 years; ICD-10 codes and NVDRS variables were used to capture deaths attributable to poisoning and maltreatment | 1850 deaths (122 attributed to poisoning) | Manual review | No | Completeness of narrative depended on parents/caregivers' willing to share details of the circumstances; human errors contributed to the data validity/comprehensiveness; NVDRS did not identify or track synthetic opioids |
| Farmer Suicide in Wisconsin: A Qualitative Analysis | Kohlbeck, 2022 | 2012 - 2016 | Suicide | To examine circumstances of suicides among farmers | Wisconsin residents who died by suicides and worked in farming-related jobs (e.g., farmer, farm manager, and farm worker) | 73 incidents | Manual review | No | Variability in the quality and detail of narrative by county; less data from women and people of color; data might not reflect unbiased view of the suicide contexts |
| Factors Contributing to Filicide-Suicide: Differences Between Male and Female Perpetrators | Murfree, 2022 | 2013 - 2015 | Filicide-suicide | To identify the contextual factors experienced by the perpetrators of filicide-suicide and explores how these factors differed by the sex of the perpetrator | Decedents of filicide-suicides, including children victims under age 18 and their caregivers (i.e., parent, stepparent, parent's partner) who died by suicides, excluding incidents with multiple perpetrators | 76 incidents | Manual review | No | Narratives were limited to the information known by C/ME and LE and/or known through prior interactions or shared by friend or family of the decedent |
| Comparing Fatal Child Abuse Involving Biological and Surrogate Parents | Presser, 2022 | 2011-2017 | Fatal child abuse (pediatric homicide) | To examine the characteristics of fatal child abuse/pediatric homicide by biological versus surrogate parents | Children younger than 18 years who died by homicides in Georgia | 425 cases (219 from fatal child abuse) | Manual review | No | Narrative data depended on the interpretations of the abstractors; not all states opted into the Child Fatality Review module, which limited accessibility of certain variables (e.g., poverty, history of violence, substance use, and incarceration) |
| Psychologist homicide victims: The national violent death reporting system and other sources | Robiner, 2022 | 2003-2018 | Homicide | To conduct preliminary exploration into homicides of psychologists and provide resources for prevention | Psychologists who died by homicides | 27 victims (12 identified using NVDRS) | Manual review | Google and internet searches used to identify additional homicides of psychologists (incident dates: 1977-2021) | Limited information on the patient-related incidents; NVDRS likely failed to accurately capture homicides among psychologists |
| Please forgive me:' Asian and Pacific Islander Americans’ suicide notes | Wong, 2022 | 2003 - 2017 | Suicide | To examine if demographics predicted whether decedents would leave a suicide note and test for moderating effects; analyze notes to test if the expression of conciliatory interpersonal communications and negative interpersonal concerns differed as a function of demographics | Non-Hispanic APIAs who died by suicide | 3657 decedents | Manual review | No | Variation in the amount of details in the narrative; C/ME and LE may have injected their own interpretation of the suicide notes; lacked data on acculturation status, sexual orientation, religious affiliation, and ethnic differences |

**Note: These challenges are those noted by the authors as being relevant to narratives or NVDRS/VDRS data in general rather than the study-specific limitations, such as limitations pertaining to the analytical approaches and generalizability of NVDRS data at the time of the study*.

**Abbreviations**

US: United States

NVDRS: National Violent Death Reporting System

VDRS: Violent Death Reporting System

NVSS: National Vital Statistics System

CDC: Centers for Disease Control

WONDER: Wide-Ranging Online Data for Epidemiologic Research

WISQARS: Web-based Injury Statistics Query and Reporting System

BRFSS: Behavioral Risk Factor Surveillance System

FBI: Federal Bureau of Investigation

NYPD: New York Police Department

C/ME: Coroner/Medical Examiner

LE: Law Enforcement

ICD: International Classification of Diseases

IPV: Intimate Partner Violence

IPH: Intimate Partner Homicide

IPP: Intimate Partner Problem

APIA: Asian and Pacific Islander American
